# Lysophosphatidic acid activates Arf6 to promote the mesenchymal malignancy of renal cancer

**DOI:** 10.1038/ncomms10656

**Published:** 2016-02-08

**Authors:** Shigeru Hashimoto, Shuji Mikami, Hirokazu Sugino, Ayumu Yoshikawa, Ari Hashimoto, Yasuhito Onodera, Shotaro Furukawa, Haruka Handa, Tsukasa Oikawa, Yasunori Okada, Mototsugu Oya, Hisataka Sabe

**Affiliations:** 1 Department of Molecular Biology, Graduate School of Medicine, Hokkaido University, Sapporo 060-8638, Japan; 2 Division of Diagnostic Pathology, Keio University Hospital, Tokyo 160-0016, Japan; 3 Department of Gastroenterological Surgery II, Graduate School of Medicine, Hokkaido University, Sapporo 060-8638, Japan; 4 Department of Pathology, Keio University School of Medicine, Tokyo 160-0016, Japan; 5 Department of Urology, Keio University School of Medicine, Tokyo 160-0016, Japan; 6 Present address: Division of Diagnostic Pathology, Keio University School of Medicine

## Abstract

Acquisition of mesenchymal properties by cancer cells is critical for their malignant behaviour, but regulators of the mesenchymal molecular machinery and how it is activated remain elusive. Here we show that clear cell renal cell carcinomas (ccRCCs) frequently utilize the Arf6-based mesenchymal pathway to promote invasion and metastasis, similar to breast cancers. In breast cancer cells, ligand-activated receptor tyrosine kinases employ GEP100 to activate Arf6, which then recruits AMAP1; and AMAP1 then binds to the mesenchymal-specific protein EPB41L5, which promotes epithelial–mesenchymal transition and focal adhesion dynamics. In renal cancer cells, lysophosphatidic acid (LPA) activates Arf6 via its G-protein-coupled receptors, in which GTP-Gα12 binds to EFA6. The Arf6-based pathway may also contribute to drug resistance. Our results identify a specific mesenchymal molecular machinery of primary ccRCCs, which is triggered by a product of autotaxin and it is associated with poor outcome of patients.

## Abstract (web summary)

Acquisition of mesenchymal properties by cancer cells is a critical event for the development of malignancy. Here, the authors show that in renal cancer cells, lysosphosphatidic acid does not utilize the RhoA pathway but specifically activates the Arf6 mesenchymal pathway via its GPCRs and EFA6 to promote invasion, metastasis and drug resistance.

## Graphical abstract

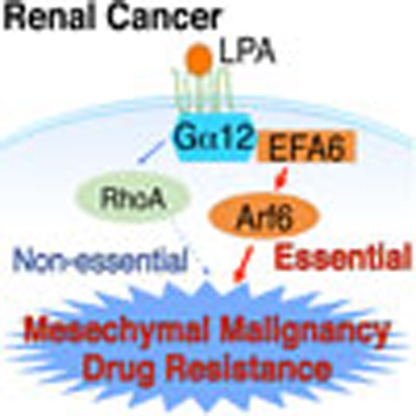

Invasion and metastasis of cancer cells, as well as their resistance to treatment, are the major causes of patient death. Clear cell renal cell carcinomas (ccRCCs) account for ∼70% of kidney cancers^1^; and metastasis occurs in about 30–40% of these patients^2^. Most ccRCCs originate from the lining epithelium of the renal tubules in the kidney^3^, which face peripheral body fluids. Acquisition of mesenchymal properties by cancer cells, even transiently, via processes resembling epithelial–mesenchymal transition is thought to be a critical event for the development of malignancy and metastasis into distant areas^4^,^5^,^6^. This may occur through the enhancement of the resistance to anoikis^7^ to maintain cell survival rates during their distant metastasis or possibly also through facilitating the motility and invasive activities of cancer cells. Moreover, the acquisition of mesenchymal properties is often coupled with the resistance of cancer cells to therapeutic drugs^6^.

Arf6, which is a small GTPase primarily regulating the recycling of plasma membrane components^8^, and its downstream effector AMAP1 (also called ASAP1 and DDEF1) are frequently overexpressed in breast cancers, and constitute a signalling pathway that promotes invasion and metastasis of cancer cells by downregulating E-cadherin-based cell–cell adhesion and upregulating recycling of β1 integrins^9^,^10^,^11^,^12^. Moreover, EPB41L5, which was originally identified as being induced during the epithelial–mesenchymal transition of mammary epithelial cells^13^, is frequently overexpressed in breast cancer cells as an integral binding partner of AMAP1 that drives invasion and metastasis (will be published elsewhere). Mechanistically, EPB41L5 binds to p120^cat^ to sequester E-cadherin from p120^cat^, and the released E-cadherin molecules are then internalized from the cell surface, leading to the disruption of epithelial cell–cell adhesions^13^. EPB41L5 also enhances focal adhesion turnover, which might promote cell motile activities^13^. In breast cancer cells, receptor tyrosine kinases (RTKs), such as epidermal growth factor receptor, activate Arf6, by recruiting the guanine nucleotide exchanger GEP100 (also called BRAG2)^11^. Clinically, robust expression of Arf6 pathway components in primary breast tumours statistically correlates with tumour malignancy and the poor overall survival of patients^10^,^11^,^14^. The Arf6 pathway appears to also exist in subpopulations of lung adenocarcinomas^15^ and head and neck cancers^16^, and statistically correlates with their metastatic recurrence and poor outcomes.

ccRCCs and breast cancers both originate mainly from cells located within epithelial ductal structures. Moreover, for both of them acquisition of mesenchymal properties are thought to be critical for malignant development. We here investigated whether ccRCCs also utilize the Arf6-based pathway for their malignancy development, including their drug resistance. Our results demonstrate that ccRCCs also frequently overexpress components of the Arf6-based mesenchymal pathway, and that this pathway is activated by G-protein-coupled receptors (GPCRs) rather than RTKs in ccRCCs. The Arf6-based mesenchymal pathway not only promotes invasion and metastasis, but crucially contributes to drug resistance. Our results identify the molecular machinery that drives the mesenchymal-type malignancy of large populations of primary ccRCCs, which is critical to the poor overall survival of patients.

## Results

### LPA activates the Arf6 mesenchymal pathway in ccRCC cells

**Figure 1:**
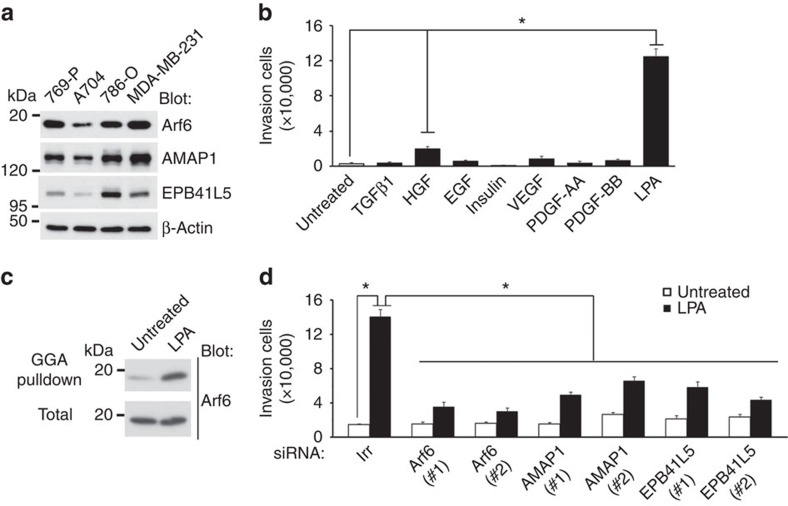
LPA activates Arf6 to promote the invasion of 786-O cells. (**a**) The expression of Arf6 pathway proteins in ccRCC cell lines, assessed by immunoblotting analysis of total cell lysates using the indicated antibodies. Lysates of MDA-MB-231 cells were included for comparison. β-actin immunoblotting is shown as a control. (**b**) Matrigel invasion activities of 786-O cells in the presence of various ligands, as indicated. (**c**) Arf6 activity of 786-O cells on stimulation by LPA. (**d**) Requirement for Arf6, AMAP1 and EPB41L5 in LPA-induced Matrigel invasion of 786-O cells, as assessed by their gene silencing using specific siRNAs. (**b**,**d**) Error bars show the mean±s.e.m., *n*=3. *P<0.01. Statistical analyses were performed using analysis of variance.

We first found that a highly invasive model cell line of ccRCCs, namely 786-O, expressed Arf6, AMAP1 and EPB41L5 at high levels, almost comparative with those observed in highly invasive breast cancer model cell line MDA-MB-231 (ref. 17; Fig. 1a). In contrast, such overexpression of all Arf6 pathway components was not observed in 769-P cells and A704 cells (Fig. 1a), which were reported to be weakly invasive^18^. Similarly to that in MDA-MB-231 cells, complex formation of AMAP1 with EPB41L5 was detected in 786-O cells (Supplementary Fig. 1).

Unlike breast cancers, there has been no clear evidence supporting that the robust expression of RTKs is statistically associated with the malignant development of ccRCCs. We found that epidermal growth factor, hepatocyte growth factor, insulin, vascular endothelial growth factor, and the AA-type and BB-type platelet-derived growth factor (PDGF) do not significantly enhance the Matrigel invasion activities of 786-O cells (Fig. 1b). Transforming growth factor β1 also did not enhance Matrigel invasion activities (Fig. 1b). By searching possible ligands, we found that lysophosphatidic acid (LPA) drastically enhanced the Matrigel invasion activity of 786-O cells, and activated Arf6 (Fig. 1b,c). Small interfering RNA (siRNA)-mediated silencing of *Arf6*, *AMAP1* and *EPB41L5* each effectively blocked the LPA-induced invasion of 786-O cells, without affecting cell viability (Fig. 1d; Supplementary Fig. 2a,b). The expression of a dominant-negative mutant of Arf6, that is, the GTP binding-defective Arf6 (T27N), in 786-O cells also inhibited LPA-induced invasion without affecting cell viability (Supplementary Fig. 2c–e). These results indicated that LPA activates Arf6 in 786-O cells to promote cell invasion activity via the Arf6-AMAP1-EPB41L5 mesenchymal pathway.

### RhoA is dispensable for LPA-induced invasion of ccRCCs

**Figure 2:**
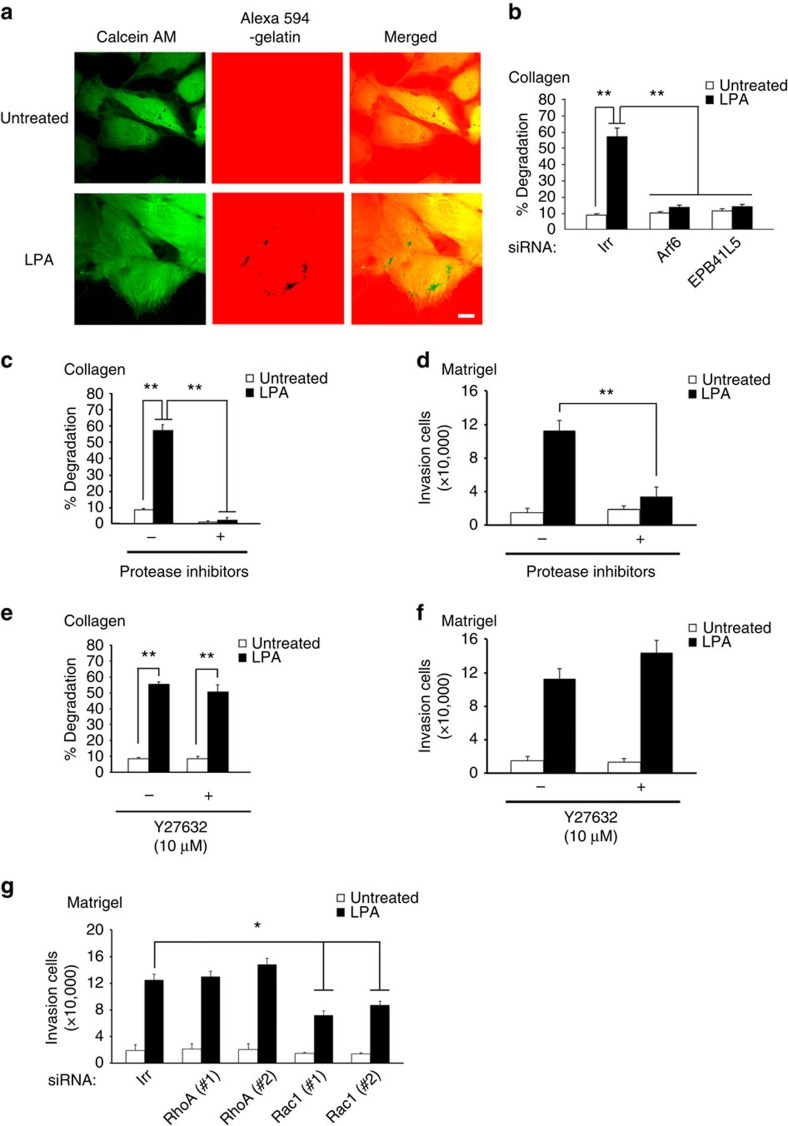
Properties of LPA-induced invasion of 786-O cells. (**a**) Invadopodia formation on LPA stimulation. Cells were visualized using calcein AM, and areas of degradation of cross-linked Alexa 594-labelled collagen gels were shown as colourless areas (black dots in the middle panel). Scale bar, 10 μm. (**b**) Involvement of Arf6 and EPB41L5 in invadopodia formation. Cells were pretreated with siRNAs for *Arf6*, *EPB41L5* or an irrelevant sequence (Irr), before being subjected to the invadopodia formation assay with or without LPA. (**c**–**f**) Protease inhibitors (**c**,**d**), but not Y27632 (**e**,**f**), block LPA-induced matrix degradation (**c**,**e**) and Matrigel invasion (**d**,**f**). Inhibitors were applied to the cells 6 h before the experiments and were present during the experiments. (**g**) RhoA, but not Rac1, is dispensable for LPA-induced invasion. Cells were pretreated with siRNAs for *RhoA*, *Rac1* or Irr, before being subjected to the Matrigel invasion assay with or without LPA. (**b**,**c**,**e**) Percentages of cells exhibiting invadopodia are shown as % degradation. (**b** and **c**–**g**) Error bars represent the mean±s.e.m., *n*=3. *P<0.05, **P<0.01. Statistical analyses were performed using analysis of variance.

LPA can also activate other small GTPases, such as RhoA and Rac1 (refs 19, 20). Moreover, some cancer cells exhibit amoeboid-type invasion, which does not generally require protease activities, such as matrix metalloproteinase (MMP)^21^. The requirement for Rho-family GTPases has also been shown to be different between the protease-dependent-type invasion and the amoeboid-type invasion^21^. We then investigated the types of invasion utilized by LPA-stimulated 786-O cells. We found that LPA-stimulated 786-O cells form invadopodia, which was assessed by the degradation of cross-linked collagen gels (Fig. 2a). We confirmed the involvement of the Arf6-based mesenchymal pathway in this invadopodia formation, by siRNA-mediated silencing of *Arf6* or *EPB41L5* (Fig. 2b). Moreover, similarly to that in breast cancer cells, Arf6 and EPB41L5 were found to accumulate at the invadopodia of LPA-stimulated 786-O cells (Supplementary Fig. 3a). Treatment of 786-O cells with a protease inhibitor cocktail^22^ blocked the LPA-induced invadopodia formation (Fig. 2c), confirming the involvement of protease activities. Matrigel invasion activity, which was used above, involves the transmigration of cells through Matrigel-coated membranes (see Methods section). The protease inhibitor cocktail also blocked the Matrigel invasion of LPA-stimulated 786-O cells (Fig. 2d). On the other hand, the ROCK inhibitor Y27632 did not notably affect LPA-induced invadopodia formation (Fig. 2e) or Matrigel invasion (Fig. 2f). It has been shown that RhoA activity is essential for amoeboid-type invasion, but not for MMP-dependent invasion^23^. Conversely, Rac1 activity may be required for MMP-dependent invasion, but is suppressed in amoeboid-type invasion^23^. Consistently, silencing of *RhoA* also did not notably affect LPA-induced Matrigel invasion activities, whereas silencing of *Rac1* significantly decreased LPA-induced invasion (Fig. 2g; Supplementary Fig. 3b,c). These results indicated that the LPA-induced invasion of 786-O cells, which is dependent on Arf6, substantially utilizes matrix degradation activities, and that RhoA activity is dispensable to this invasion.

### The Arf6 mesenchymal pathway generates high invasiveness

We then sought to reconstitute the Arf6-based pathway in weakly invasive ccRCC cells. 769-P cells expressed Arf6 and AMAP1 at levels comparable to those in 786-O cells, whereas this cell line expresses EPB41L5 at a marginal level (Fig. 1a). We found that the exogenous overexpression of EPB41L5, tagged with hemagglutinin (HA) in 769-P cells significantly enhanced the LPA-induced invasion activity (Supplementary Fig. 4a–c). Therefore, it is likely that the Arf6-based mesenchymal pathway has the potential to promote the invasion of different ccRCC cells in response to LPA.

### LPA receptors are responsible to Arf6 activation in invasion

**Figure 3:**
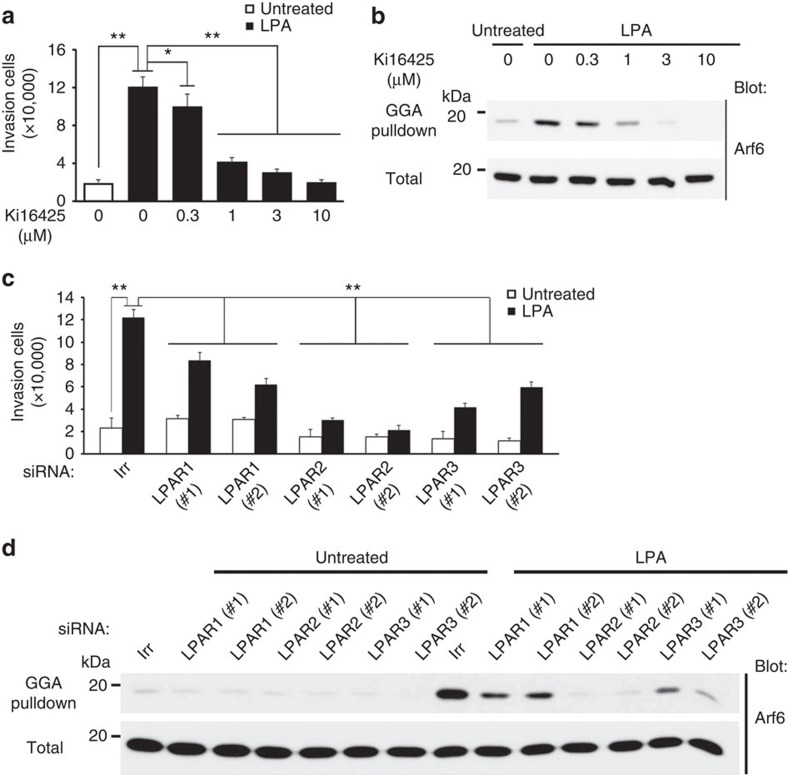
Involvement of LPARs in LPA-induced invasion and Arf6 activation of 786-O cells. (**a**,**b**) Ki16425 inhibits LPA-induced Matrigel invasion activity (**a**) and Arf6 activation (**b**). Ki16425 was applied to the cells at the indicated concentrations, 3 h before the experiments and was present during the experiments. (**c**,**d**) Involvement of endogenous LPAR1-3 in LPA-induced Matrigel invasion (**c**) and Arf6 activation (**d**), as assessed by their gene silencing using specific siRNAs, as indicated. In **c** and **d**, siRNA with an irrelevant sequence (Irr) was included as a control. (**a**,**c**) Error bars show the mean±s.e.m., *n*=3. *P<0.05, **P<0.01. Statistical analyses were performed using analysis of variance.

LPA binds to LPA receptor (LPAR) types 1–6, which are GPCRs, and possibly also to peroxisome proliferator-activated receptor-γ^24^. Ki16425, a low-molecular weight inhibitor of type 1, 2 and 3 LPARs^24^, blocked the LPA-induced Arf6 activation of 786-O cells, as well as their invasion in a dose-dependent manner, without affecting cell viability (Fig. 3a,b; Supplementary Fig. 5a). Silencing of these LPARs by their specific siRNAs each blocked LPA-induced Arf6 activation and cell invasion to some extent without affecting cell viability, in which silencing of LPAR2 appeared to be most effective (Fig. 3c,d; Supplementary Fig. 5b,c). Therefore, subsets of LPARs appear to be responsible for Arf6 activation and cell invasion on LPA stimulation of 786-O cells.

### EFA6A-C activate Arf6 under LPARs

**Figure 4:**
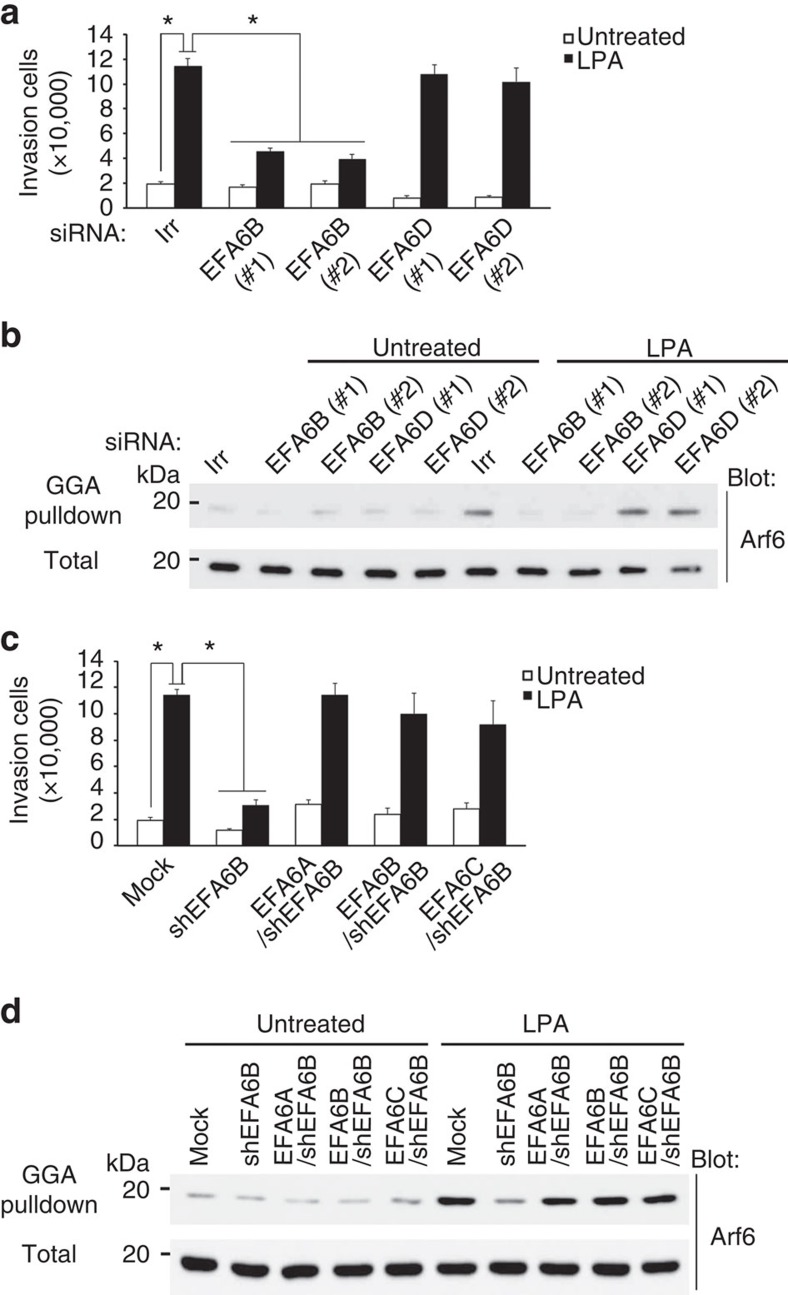
Involvement of EFA6A-C but not EFA6D in LPA-induced invasion and Arf6 activation of 786-O cells. (**a**,**b**) Requirement for endogenous EFA6B, but not endogenous EFA6D, in LPA-induced Matrigel invasion (**a**) and Arf6 activation (**b**), as assessed by their gene silencing using specific siRNAs, as indicated. (**c**,**d**) EFA6A and EFA6C, expressed in 786-O cells instead of the endogenous EFA6B (EFA6A/shEFA6B cells and EFA6C/shEFA6B cells, respectively) promoted Matrigel invasion (**c**) and Arf6 activation (**d**) in response to LPA. shEFA6B cells and EFA6B/shEFA6B cells were included as controls. (**a**,**b**) siRNA with an irrelevant sequence (Irr) was included as a control. (**a**,**c**) error bars show the mean±s.e.m., *n*=3. *P<0.01. Statistical analyses were performed using analysis of variance.

GEP100 is responsible for the activation of Arf6 under RTKs. Silencing of *GEP100* in 786-O cells did not notably affect LPA-induced Arf6 activation and cell invasion, or cell viability (Supplementary Fig. 6a–d). Inhibition of the cytohesin family members of ArfGEFs that activate Arf6, by SecinH3 (ref. 25) also did not block LPA-induced invasion (Supplementary Fig. 6e). 786-O cells also expressed other ArfGEFs, such as EFA6B and EFA6D (Supplementary Fig. 6f). Silencing of *EFA6B*, but not *EFA6D*, blocked LPA-induced Arf6 activation and cell invasion, without affecting cell viability (Fig. 4a,b; Supplementary Fig. 6g,h). The EFA6 family consists of four members, namely EFA6A-D^26^. EFA6A and EFA6C, when expressed in 786-O cells instead of the endogenous *EFA6B*, also activated Arf6 and promoted cell invasion in response to LPA (Fig. 4c,d; Supplementary Fig. 6i). Therefore, all EFA6 family members except for EFA6D appear to have the potential to activate Arf6 on LPA stimulation to promote cell invasion activity.

### The GTP-bound form of Gα12 binds to EFA6

**Figure 5:**
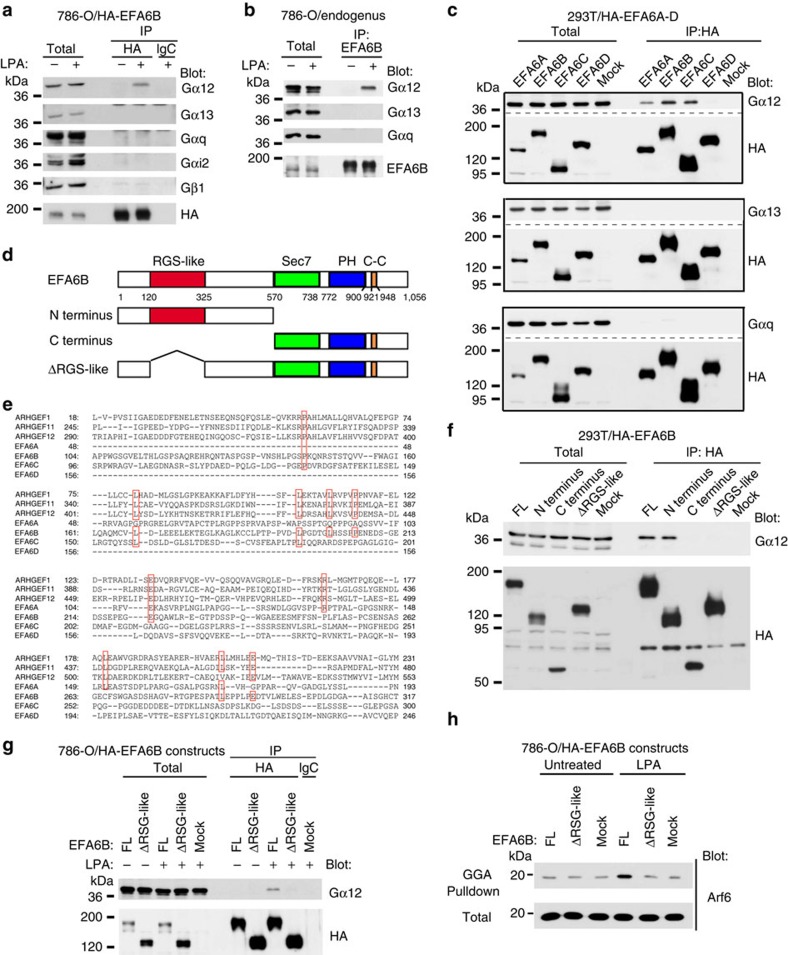
GTP-Gα12 bind to EFA6A-C but not to EFA6D to activate Arf6. (**a**) Binding of HA-EFA6B with Gα12 on LPA stimulation. EFA6B/shEFA6B cells (786-O/HA-EFA6B) were either stimulated with LPA or left untreated for 5 min, and the co-precipitation of endogenous trimeric G-proteins with HA-EFA6B was then analysed by anti-HA immunoprecipitation (IP) coupled with immunoblotting using the indicated antibodies. An irrelevant mouse IgG (IgG) was used as a control. (**b**) Endogenous association of Gα12 with EFA6B. Co-precipitation of Gα12, but not Gα13 or Gαq, with anti-EFA6B immunoprecipitants (IP) of 786-O cell lysates was examined, coupled with immunoblotting as indicated. (**c**) EFA6A-C, but not EFA6D, binds to GTP-Gα12. 293T cells were transfected with plasmids encoding HA-EFA6A-D or with an empty vector (Mock), together with plasmids encoding Gα12QL, Gα13QL, or GαqQL; and co-precipitation of these G-proteins with EFA6 family members was assessed as in **a**. (**d**) Schematic representation of the domain structure of EFA6B and its mutants. (**e**) The primary structures of the RGS domains of RhoGEFs, ARHGEF1, ARHGEF11, and ARHGEF12, and their amino acid alignment with the N-terminal regions of EFA6A-D. Conserved amino acids are marked in red boxes. (**f**) The RSG-like domain is essential for the binding to GTP-Gα12. Full-length HA-EFA6B (FL) and its mutants were expressed in 293T cells together with Gα12QL, as indicated, and their co-precipitation was assessed as in **a**. An empty vector (Mock) was included as a control. (**g**,**h**) Requirement for the RSG-like domain in the binding to endogenous Gα12 and the activation of Arf6. Full-length HA-EFA6B (FL) and its ΔRGS-like mutant were expressed in 786-O cells, in which endogenous EFA6B was silenced; and co-precipitation of HA-EFA6B proteins with Gα12 (**g**) and activation of Arf6 (**h**) were examined on LPA stimulation. (**a**–**c**,**f**–**h**) Total, total cell lysates.

GTP-bound Gα subunits and Gβγ heterodimers are released on the activation of GPCRs, and bind to their cognate partners to transmit signalling events. We expressed EFA6B, tagged with a HA-tag, in 786-O cells, and found that Gα12, but not Gα13, Gαq, Gαi2 or Gβ1, is coprecipitated with this protein on LPA stimulation (Fig. 5a). Endogenous complex formation of EFA6B with Gα12, but not with Gα13 or Gαq, in 786-O cells was confirmed (Fig. 5b). Consistently, Gα12QL, a GTP-bound form of the Gα12 mutant, but not Gα12GA (a GTP-binding-defective mutant of Gα12), Gα13QL or GαqQL, clearly coprecipitated with HA-EFA6B when they were coexpressed in 293T cells by complementary DNA (cDNA) transfection (Fig. 5c; Supplementary Fig. 7a). The expression of Gα12QL in 786-O cells induced the activation of Arf6 in the absence of LPA stimulation (Supplementary Fig. 7b). These cells also exhibited slightly, but significantly, augmented invasion activity without LPA stimulation, as compared with the parental cells (Supplementary Fig. 7c). In this regard, it should be noted that although efficient cell invasion requires the directional activation of cell surface receptors, such an exogenous expression of Gα12QL by cDNA transfection might have caused the non-directed activation of GPCR signalling at the cell surface. Furthermore, we found that Gα12QL was also coprecipitated with EFA6A and EFA6C, but not with EFA6D (Fig. 5c). Together with the above results, these results indicated that when Gα12 is activated, it is engaged in the activation of Arf6 under LPARs, via its potential to physically interact with EFA6 family members, except for EFA6D.

### The RGS-like domain mediates binding of EFA6A-C with Gα12

It has not been clarified to date whether ArfGEFs bind to the Gα subunits, whereas it is known that certain types of RhoGEFs bind to them^27^. p115-RhoGEF, PDZ-RhoGEF and LARG3 each contains the regulator of G-protein signalling (RGS) domain that mediates their binding to Gα12/13 (ref. 27). EFA6 consists of the Sec7 domain, the PH domain and a coiled-coil region, but not the RGS domain (Fig. 5d). However, alignment of the primary structures of the N-terminal regions of EFA6A, EFA6B and EFA6C with the RGS domain revealed several conserved amino acids (Fig. 5e). Notably, the N-terminal region of EFA6D is significantly different from those of EFA6A-C, and lacks these common amino acids (Fig. 5e). We generated the N-terminal half of EFA6B containing the ‘RGS-like' region fused to the HA-tag, and found that this construct binds to Gα12QL, as in the case of the full-length EFA6B construct (FL), whereas a construct of the C-terminal half and that with a deletion in the RGS-like region (ΔRGS-like) did not (Fig. 5d,f). When the ΔRGS-like mutant of EFA6B was expressed in 786-O cells in which endogenous EFA6B was silenced, it neither bound to Gα12 nor activated Arf6 on LPA stimulation (Fig. 5g,h). Therefore, the RGS-like region found in EFA6A-C, but not in EFA6D, appears to be primarily responsible for interaction with the active form of Gα12 to activate Arf6.

### LPAR2 and the Arf6 mesechymal pathway promote metastasis

**Figure 6:**
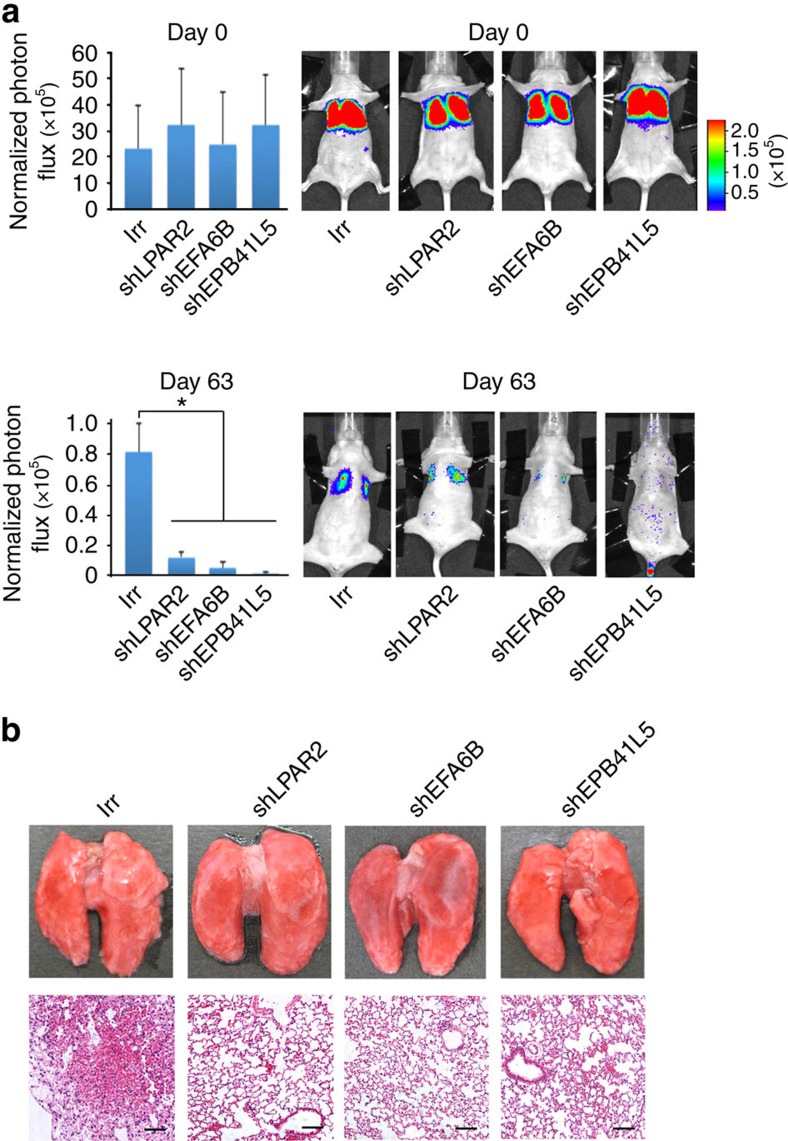
Requirement for LPAR2 and the Arf6 mesechymal pathway in the lung metastasis. 786-O cells expressing a luciferase reporter gene and shRNA plasmids, each targeting *EPB41L5* (shEPB41L5), *EFA6B* (shEFA6B) or *LPAR2* (shLPAR2), were injected into tail veins of nude mice. Cells treated with shRNA plasmids bearing an irrelevant sequence (Irr) were included as a control. (**a**) Bioluminescence intensities of the chests of the mice, measured on day 0 and day 63, are shown. Results are shown as means±s.d., *n*=5. *P<0.01. Representative bioluminescence images of mice are shown on the right. Statistical analyses were performed using analysis of variance. (**b**) Representative images of lungs on day 63 post injection. Images of whole lungs (top) and hematoxylin and eosin staining of sections (bottom). Scale bar, 100 μm.

We have previously shown that GEP100 and AMAP1 are critical for the metastasis of breast cancer cells *in vivo*^10^,^11^. Likewise, we here demonstrate that the silencing of *LPAR2*, *EFA6B* and *EPB41L5* by their specific short hairpin RNAs (shRNAs) each effectively blocks the metastasis of 786-O cells into the lungs of nude mice, in which cells were originally injected into tail veins (Fig. 6a,b; Supplementary Fig. 8a,b). Silencing of these genes did not affect cell growth measured *in vitro* (Supplementary Fig. 8c). Inhibition of Matrigel invasion activities by these shRNA treatments was also confirmed *in vitro* (Supplementary Fig. 8d). On the other hand, because silencing of *Arf6* in 786-O cells for longer than a day significantly affected cell growth *in vitro* (Supplementary Fig. 8e,f), we were unable to investigate the precise effects of *Arf6* silencing on metastasis.

### The Arf6 mesenchymal pathway contributes to drug resistance

**Figure 7:**
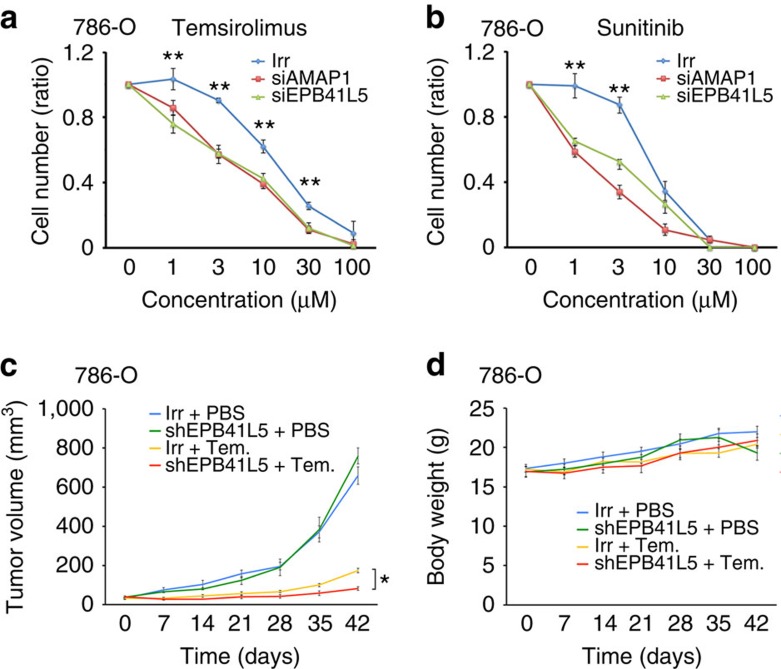
The Arf6-based mesenchymal pathway in drug resistance. (**a**,**b**) Silencing of AMAP1 and EPB41L5 enhances the drug sensitivity of 786-O cells. 786-O cells, pretreated with siRNA duplexes for *AMAP1* or *EPB41L5*, or with an irrelevant sequence (Irr), were treated with Temsirolimus (**a**) or Sunitinib (**b**) at the indicated concentrations for 3 days, and numbers of viable cells were then analysed. Data are presented as ratios, by normalizing values obtained by setting the untreated cells of each siRNA treatment as 1.0. Error bars show the mean±s.e.m., *n*=3. *P<0.05, **P<0.01. Statistical analyses were performed using analysis of variance. (**c**,**d**) 786-O cells treated with shRNAs for *EPB41L5* (shEPB41L5) or an irrelevant sequence (Irr), were subcutaneously injected into nude mice. Ten days after injection, mice were randomized into two groups, those treated with Temsirolimus (10 mg kg^−1^) (+Tem.) and those treated with PBS (+PBS) on a QDx5 schedule, and their tumour sizes (**c**) and body weights (**d**) were monitored for further 6 weeks, as indicated. Error bars show the mean±s.e.m., *n*=6. *P<0.05. Statistical analyses were performed using Student's *t*-test.

Resistance of cancer cells to therapeutic drugs is a major clinical problem. We next tested whether the Arf6-based mesenchymal pathway is involved in the drug resistance of ccRCCs. Temsirolimus is a currently used inhibitor of mTOR activity^28^. Sunitinib is a multi-kinase inhibitor, although it may primarily affect the tumour microenvironment^28^,^29^. Silencing of *AMAP1* and *EPB41L5* in 786-O cells each significantly reduced cell survival on treatment with these drugs *in vitro* (Fig. 7a,b), whereas silencing of these genes on their own did not notably affect the viability of 786-O cells, as shown above. We also demonstrated that silencing of *EPB41L5* in 786-O cells significantly reduced their growth *in vivo* (that is, tumour volume) on treatment with Temsirolimus, as compared with the control Temsirolimus-treated 786-O cells, whereas this silencing did not notably affect the growth of 786-O cells in mice in the absence of the drug (Fig. 7c). A decrease in body weight was not observed under these conditions (Fig. 7d). On the other hand, silencing of *AMAP1* and *EPB41L5* in 769-P cells and A704 cells did not further reduce their survival on treatment with these drugs, as compared with the similarly treated parental cells (Supplementary Fig. 9a–d). Therefore, it is likely that the existence of all components of the Arf6-based mesenchymal pathway at high levels, but not their solitary overexpression in the absence of the intact Arf6-based pathway, appears to contribute to the drug resistance of ccRCCs, although we do not yet clearly understand the molecular mechanisms by which the Arf6-based mesenchymal pathway can contribute to drug resistance.

### The Arf6 mesenchymal pathway and poor outcome of patients

**Figure 8:**
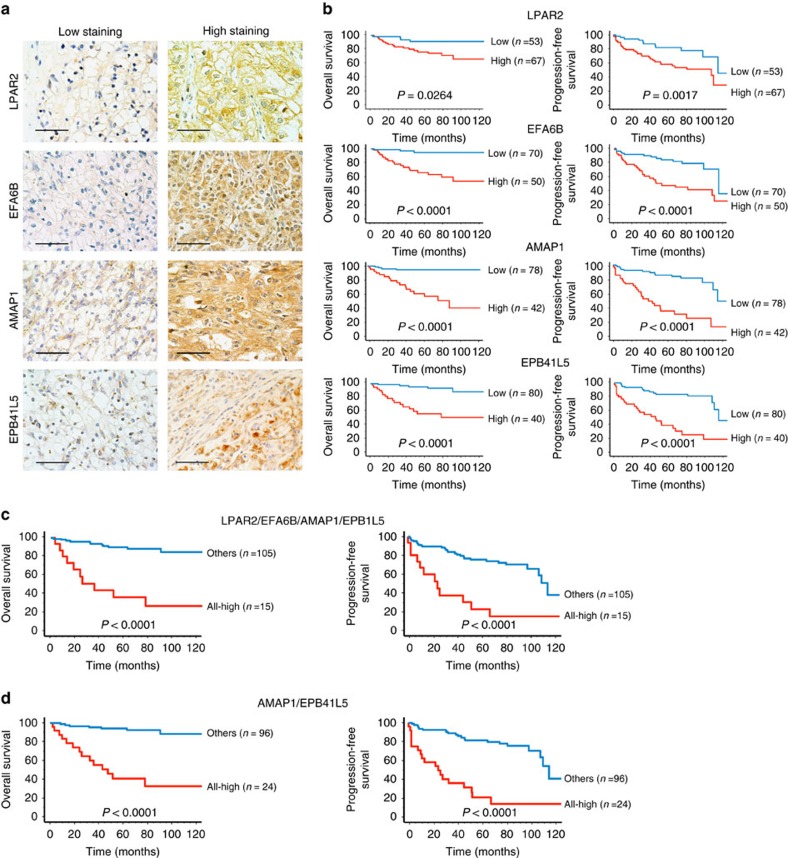
The Arf6-based mesenchymal pathway in poor clinical outcome of ccRCC patients. (**a**) Representative images of immunohistochemical staining of LPAR2, EFA6B, AMAP1 and EPB41L5 in human primary ccRCC specimens. Scale bars, 50 μm. (**b**–**d**) Kaplan–Meier curves of the overall survival and progression-free survival of ccRCC patients (*n*=120), with regard to the expression levels of each single component of the Arf6 pathway (**b**), with regard to the high expression of all components (**c**), and with regard to the simultaneous high expression of AMAP1 and EPB41L5 (**d**), as indicated. *P* values represent the results of the log-rank test.

To obtain clinical relevance for the results obtained in our study, we finally investigated whether robust expression of the Arf6-based mesenchymal pathway statistically correlates with the poor outcome of ccRCC patients. We examined the expression of LPAR2, EFA6B, AMAP1 and EPB41L5 by immunohistochemistry in 120 specimens of primary ccRCCs, each from a different patient, while we were unable to analyze Arf6 owing to the lack of an antibody applicable to immunohistochemistry. The clinicopathological characteristics of the patients at the time of nephrectomy are summarized in Supplementary Tables 4 and 5. By classifying the specimens by the expression levels of each of these proteins (see Fig. 8a for representative staining images, and Methods section for details of the scoring), we found that high expression (that is, high staining) of these proteins statistically correlates with the poor overall survival and poor disease-free survival of the patients, in which simultaneous high expression of all of these proteins exhibited the highest correlation (Fig. 8b,c). We also found that the simultaneous high expression of AMAP1 and EPB41L5 (AMAP1-high/ EPB41L5-high) provides an accurate biomarker that tightly correlates with the poor outcome of the patients (Fig. 8d). These conclusions were unaffected even when we only analysed patients who did not have metastasis at the time of diagnosis (pM0, *n*=110; Supplementary Fig. 10a,b). Therefore, our results indicated that the robust expression of components of the Arf6 pathway in primary ccRCCs provides excellent biomarkers predictive for the poor outcomes of patients. Intratumour heterogeneity may exist in renal cancers^30^,^31^, which seemed to be reflected by the non-uniform expression of the EPB41L5 protein, even within the same cancerous lesion (Fig. 8a). On the other hand, LPAR2, EFA6B and AMAP1 proteins appeared to be expressed rather uniformly within each lesion, although expression levels differed among different lesions (Fig. 8a).

## Discussion

Our results described in this paper revealed that LPA directly activates the small GTPase Arf6 via its GPCRs and EFA6 in ccRCCs to promote mesenchymal-type invasion, metastasis and also drug resistance; in which AMAP1, and its mesenchymal-type binding partner EPB41L5 are also essential. We, moreover, provided evidence supporting that high expression levels of components of the Arf6-based mesenchymal pathway, as well as LPAR2 are tightly correlated with the poor overall survival of patients.

LPA has been well recognized as a potent activator of Rho^19^, as well as Rac^20^ and several other intracellular signalling pathways, and has long been believed to thereby promote tumour malignancy^32^. Our results clearly show that LPA has another basic function in promoting tumour malignancy, via the activation of Arf6. Our results have, moreover, shown that the LPA-induced invasiveness of renal cancer cells appears to be protease-dependent, but does not appear to require RhoA, similarly to previous reports on the MMP-dependent mesenchymal invasiveness of melanomas^23^. Our findings are thus intriguing because most ccRCCs originate from the proximal convoluted tubules of the kidneys, which face body fluids; and because high amounts of LPA may easily be produced extracellularly by the activity of autotaxin from lysophosphatidylcholine, which is abundant in body fluids^24^. Autotaxin, also known as ectonucleotide pyrophosphatase/phosphodiesterase 2, was originally identified as an autocrine factor secreted by melanomas to stimulate their cell motility^33^, and is now known to be expressed extracellularly by different cancer cells at high levels, including ccRCCs^34^. We showed that the presence of the Arf6-based mesenchymal pathway in primary ccRCCs may be easily predicted by immunostaining of proteins such as AMAP1 and its mesenchymal-specific partner EPB41L5; and moreover, we have shown the Arf6-based mesenchymal pathway provide excellent molecular therapeutic targets to block cancer metastasis and to enhance cancer sensitivity to therapeutic drugs (refs 10, 11, 12, 35, and this paper). Thus, it awaits to be clarified as to whether circulating tumour cells, as well as tumours at metastatic sites in ccRCCs patients frequently express these proteins of the Arf6-based mesenchymal pathway at high levels.

## Methods

### Cells

786-O, 769-P, A704, MDA-MB-231 and 293T cells were purchased from ATCC. These cells were not listed by the International Cell line Authentication Committee (ICLAC) as misidentified cell lines (3 October 2014). Renal carcinoma cell lines were cultured at 37 °C in RPMI 1640 (Invitrogen) supplemented with 10% FCS (HyClone). MDA-MB-231 cells were maintained in a 1:1 mixture of DMEM (Invitrogen) and RPMI 1640 supplemented with 10% FCS and 5% Nu-Serum (BD Biosciences), as described previously^11^. 293T cells were cultured in DMEM supplemented with 10% FCS. 293FT cells were purchased from Invitrogen, and cultured at 37 °C according to the manufacturer's instructions. Plat-E cells were a gift from Dr Kitamura (Tokyo University), and were cultured at 37 °C in DMEM containing 10% FCS. All cell lines were determined to be free of mycoplasma using cytochemical staining of DNA with 4',6-diamidino-2-phenylindole (Sigma-Aldrich). No antibiotics were used in our cell cultures to avoid latent infection of mycoplasma to cultured cells.

### Ligand stimulation

Cells were starved for 12 h with RPMI supplemented with 0.5% FCS, before stimulation by ligands. Ligands used were LPA (5 nM, Santa Cruz), transforming growth factor β1 (2 ng ml^−1^, R&D Systems), hepatocyte growth factor (10 ng ml^−1^, PeproTech), epidermal growth factor (10 ng ml^−1^, PeproTech), vascular endothelial growth factor (10 ng ml^−1^, PeproTech), PDGF-AA (50 ng ml^−1^, PeproTech), PDGF-BB (50 ng ml^−1^, PeproTech) and insulin (5 ng ml^−1^, Sigma-Aldrich). For the Matrigel invasion assay, ligands were added throughout the assay. For measurement of Arf6 activity, pre-starved cells were stimulated by ligands for 5 min before analyses. Cell viabilities were measured using Cell Counting Kit-8 (Dojindo Molecular Technologies), according to the manufacturer's instructions.

### Chemicals

Temsirolimus (PZ0020) and Sunitinib (PZ0012) were purchased from Sigma-Aldrich. Ki16425 (355025-24-0) and Y27632 (257-00511) were from Wako Chemicals, and SecinH3 was from Santa Cruz (853625-60-2). Other chemicals were purchased from Wako Chemicals, unless otherwise indicated. Since Y27632 did not inhibit cell invasiveness in our experiments, which used renal cancer cells, we confirmed the activity of Y27632 by its inhibition of the amoeboid-type invasion of HT1080 cells, as described previously^22^.

### Antibodies and immunoblotting analyses

Affinity-purified rabbit polyclonal antibodies against human GEP100 and human AMAP1, and mouse polyclonal antibodies against human EFA6B were as described previously^10^,^11^. Rabbit polyclonal antibodies against human EPB41L5 were generated using a GST-fused peptide corresponding to amino acids (aa) 541–733. The resulting sera were first adsorbed with GST protein, and then affinity-purified using the antigen peptides. Other antibodies were purchased from the following commercial sources: mouse monoclonal antibodies against Arf6 (1:1,000, Santa Cruz), AMAP1 (1:2,000, Santa Cruz), HA-tag (1:1,000, Covance) and β-actin (1:5,000, UBI); and rabbit polyclonal antibodies against Gα12 (1:1,000, Santa Cruz Biotechnology), Gα13 (1:1,000, Sigma-Aldrich), Gαq (1:1,000, Santa Cruz Biotechnology), Gαi2 (1:1,000, Santa Cruz Biotechnology), Gβ1 (1:1,000, Upstate Biology), EFA6B (1:1,000, Sigma-Aldrich) and EFA6D (1:1,000, Santa Cruz). Donkey antibodies against rabbit and mouse IgG, each conjugated with horseradish peroxidase, were from Jackson ImmunoResearch Laboratories. Immunoblotting analysis was performed using the ECL kit (GE Healthcare), as described previously^11^.

Uncropped images of immunoblot are provided in Supplementary Figs 11–13.

### Plasmids

cDNAs encoding full-length human *EFA6A-D*; and cDNA fragments of *EFA6B* encoding the N terminus (aa 1–570), the C terminus (aa 571–1,056) and the RGS-like domain-deleted mutant (Δ121–324aa) were amplified using PCR from first-strand cDNAs prepared from human fetal brain mRNAs (Clontech). A cDNA of full-length human EPB41L5 was amplified by PCR from the first-strand cDNA of HUVEC cells. These cDNAs were ligated into the *Not*I site of pCX4-bsr^36^, in which a synthesized HA fragment was inserted into the *Bam*HI/*Eco*RI site. psd44-puro-based plasmids encoding constitutively active mutants of Gα12 (Gα12QL), Gα13 (Gα13QL) and Gαq (GαqQL) were purchased from Addgene (46825, 46828 and 46826, respectively). A cDNA encoding the dominant-negative mutant of Gα12, namely, Gα12G230A (Gα12GA), was generated by PCR and ligated into the *Bam*HI/*Nhe*I site of the psd44-puro plasmid. HA-tagged dominant-negative Arf6 (T27N) cDNA in the pcDNA3 vector was as described previously^9^. Oligonucleotides used for the PCR reactions are summarized in Supplementary Table 1.

### RNA interference and transfection

For transient siRNA-mediated gene silencing, cells were transfected with 50 nM each of the corresponding siRNA oligonucleotide duplexes (Japan BioService) using Lipofectamine RNAi Max (Invitrogen), according to the manufacturer's instructions. Two different sequences were used for each target, unless otherwise described. The siRNAs used for the knockdown of Rac1 and RhoA were from Life Technologies (for Rac1, 4390824-s11711, 4390824-s11712; for RhoA, 4390824-s758, 4390824-s759). Oligonucleotide duplexes bearing an irrelevant sequence were from Dharmacon. For stable shRNA-mediated gene silencing, pLKO.1-puro-based recombinant lentiviruses were generated. In brief, shRNAs were constructed in pLKO.1-puro from the Sigma Mission shRNA library (for LPAR2, TRCN0000011375; for EFA6B, TRCN0000427083; for EPB41L5, TRCN0000130203; for Arf6, TRCN0000294069, TRCN0000048003, Sigma-Aldrich) and a control scramble shRNA in pLKO.1-puro (1864, Addgene) were transfected into 293FT cells, together with the envelope plasmid pMD2.G (12259, Addgene) and the packaging plasmid psPAX2 (12260, Addgene), using Lipofectamine LTX (Invitrogen) according to the manufacturer's instructions. Forty-eight hours after transfection, the culture supernatants were harvested, filtered through 0.45 μm filters (Advantec), and the resultant lentivirus preparations were then applied onto target cells in the presence of Polybrene (8 μg ml^−1^). After 24 h, 4 μg ml^−1^ puromycin was added to the culture for 1 week to select infected cells. 786-O cells, in which exogenous EFA6 family members or their mutants are expressed instead of the endogenous EFA6B, were generated as follows. Cells were first transfected with an shRNA construct specific to the 3′-UTR of the *EFA6B* mRNA in pLKO.1-puro (TRCN0000427083, Sigma-Aldrich), and subjected to the selection of transfected cells. They were then infected with pCX4-bsr-based recombinant retroviruses using Lipofectamine LTX (Invitrogen), each encoding HA-tagged full-length EFA6 family members and the EFA6B mutants, which were generated using Plat-E cells and pGP-Ampho and pE-Ampho plasmids (Takara). After 24 h, infected cells were selected by the addition of 5 μg ml^−1^ Blasticidin S (Invitrogen) for at least 1 week. Target nucleotide sequences used in these experiments are summarized in Supplementary Table 2. For the constitutive overexpression of EPB41L5, 769-P cells were infected with pCX4-bsr-based recombinant retroviruses, as described above, and selected by the addition of 2 μg ml^−1^ Blasticidin S for at least 1 week. For the stable expression of Gα12QL, 786-O cells were infected with psd44-puro-based recombinant lentiviruses, as described above, and selected by the addition of 4 μg ml^−1^ puromycin for at least 1 week. For the transient overexpression of dominant-negative mutant of Arf6, 5 × 10^5^ cells were transfected with 3 μg of HA-tagged Arf6 (T27N) cloned in the pcDNA3 vector using Lipofectamine LTX (Invitrogen), and incubated for 12 h in growth medium before being subjected to analyses. To assess the efficiencies of gene silencing, total RNAs were first prepared from cells using Qiagen RNeasy Mini Kit (Qiagen), and then reverse-transcribed by M-MLV Reverse Transcriptase using oligo dT primers (Promega) at 42 °C for 60 min. These cDNAs were then subjected to 35 cycles of PCR amplification, each consisting of 95 °C for 2 min, 95 °C for 30 s, and then 50 °C (for *LPAR1* and *LPAR2*), 51 °C (for *EFA6D* and *LPAR3*), 53 °C (for *EFA6B*) or 57 °C (for *EFA6A* and *EFA6C*) for 30 sec, and finally 72 °C for 30 s. Amplified products were analysed by agarose gel electrophoresis. Primers used for the PCR reactions are summarized in Supplementary Table 3.

### Matrigel invasion

The Matrigel chemoinvasion assay was performed using Biocoat Matrigel chambers (BD Biosciences), as described previously^11^. Briefly, 1 × 10^5^ cells were seeded on the upper wells, and after 20 h they were fixed in 4% paraformaldehyde for 20 min at 25 °C. The number of cells that migrated out to the lower surface of the chamber membranes was then scored by staining with 1% crystal violet. Data were collected from three independent experiments, each performed in duplicate.

### Invadopodia formation

Invadopodia formation assays were performed as described previously^9^,^10^. In brief, 786-O cells were transfected with siRNAs targeting Arf6 or EPB41L5. Twenty-four hours after transfection, cells were plated onto a culture dish coated with Alexa 594-labelled gelatin film, and cultured for 16 h. Cells were then stained with a membrane-permeable dye, calcein AM (Invitrogen). Immunostaining of fixed cells was performed as described previously^9^,^10^. HA-EPB41L5 and Arf6-HA were visualized using an anti-HA antibody coupled with an Alexa 488-conjugated anti-mouse IgG antibody (Jackson ImmunoResearch). The number of cells degrading the gelatin film was counted using a confocal laser-scanning microscope (Model A1R, Nikon). Inhibition of extracellular proteolytic activity was performed using the following protease inhibitor cocktail^22^: GM6001 (50 μM; sc-203979, Santa Cruz Biotechnology), E64 (250 μM; E3132, Sigma-Aldrich), pepstatin A (100 μM; P5318, Sigma-Aldrich), leupeptin (2 μM; L9783, Sigma-Aldrich) and aprotinin (2.2 μM; A4529, Sigma-Aldrich).

### Arf6 activities

Arf6 activities were measured using the GST-GGA-pulldown method, as previously described^11^. Briefly, cells were harvested and washed with PBS, and then lysed in a GGA-pulldown solution (50 mM Tris-HCl (pH 8.0), 100 mM NaCl, 1 mM MgCl_2_, 0.1% SDS, 0.5% sodium deoxycholate, 1% Triton X-10, 10% glycerol, protease inhibitors (Roche), and phosphatase inhibitors (Sigma-Aldrich) at 4 °C for 10 min. After clarifying by centrifugation, 500 μg of each cell lysate was incubated with 20 μg of GST-GGA3 bound to glutathione-Sepharose beads (Amersham Pharmacia) at 4 °C for 45 min. After washing with the GGA-pulldown solution three times, beads were resuspended in Laemmli buffer and boiled for 5 min, and eluted proteins were analysed on SDS–PAGE (15% gel).

### Protein interactions

*In vivo* protein binding was assessed by the co-immunoprecipitation assay, as described previously^10^. Briefly, for analysis of EFA6B binding to the endogenous trimeric G-proteins in 786-O cells on LPA stimulation, LPA-stimulated 786-O cells, or 786-O cells expressing HA-EFA6B instead of the endogenous EFA6B, were first lysed in NP-40 buffer (1% NP-40, 150 mM NaCl, 20 mM Tris-HCl (pH 7.4), 5 mM EDTA, 1 mM Na_3_VO_4_, 1 mM PMSF, 5 μg ml^−1^ aprotinin, 2 μg ml^−1^ leupeptin, and 3 μg ml^−1^ pepstatin A) for 10 min at 4 °C. After clarifying by centrifugation, 5 mg of 786-O cell lysate was incubated with an anti-EFA6B mouse polyclonal antibody, or 750 μg of cell lysate from 786-O cells expressing HA-EFA6B instead of the endogenous EFA6B was incubated with an anti-HA mouse monoclonal antibody or an irrelevant IgG, coupled with Protein G-Sepharose beads for 1 h at 4 °C. After washing extensively, proteins precipitated with the beads were analysed on SDS–PAGE (8% gel), coupled with immunoblotting. For analysis of the possible binding of EFA6 family members with active forms of the trimeric G-proteins, 5 × 10^5^ 293T cells were transfected with 5 μg of pCX4-based plasmids each encoding an EFA6 family member or an EFA6B mutant, tagged with the HA-tag, together with 1 μg of psd44-based plasmid encoding either Gα12QL, Gα13QL, GαqQL or Gα12GA, using Polyfect (Qiagen). After incubating for 36 h, cells were lysed in NP-40 buffer, and the co-precipitation of proteins was analysed using an anti-HA antibody, as described above. For the analysis of EPB41L5-mediated complex formation of AMAP1, LPA-stimulated 786-O cells were lysed in NP-40 buffer, as described above. Cell lysate of 2.5 μg was incubated with an anti-AMAP1 mouse monoclonal antibody, coupled with Protein A-Sepharose beads for 1 h at 4 °C. After washing, proteins precipitated with the beads were analysed on SDS–PAGE (8% gel), coupled with immunoblotting.

### Metastasis assay

Immunodeficient mice (BALB/c AJc1-nu/nu, 4-week-old females) were obtained from CLEA Japan. All animal experiments were conducted under a protocol approved by the animal care committee of Hokkaido University. 786-O cells were first lentivirally infected with pLenti CMV V5-Luc blast (21474, Addgene). After selection of luciferase-positive cells using 5 μg ml^−1^ Blasticidin S, the cells were transfected with pLKO.1-puro-based shRNA plasmids each targeting *EPB41L5*, *EFA6B* or *LPAR2*, or a control-scrambled shRNA vector, described above. The transfected cells were then selected using 4 μg ml^−1^ puromycin for at least 1 week, and 2 × 10^6^ of these cells were then injected into the lateral tail vein of each female mouse at 5 weeks of age. For bioluminescence imaging, mice were anaesthetized with 3% isoflurane and then administered 150 mg kg^−1^ D-luciferin (Promega) in PBS by intraperitoneal injection. Ten minutes after the injection, bioluminescence was detected using a photon imaging system (IVIS Spectrum, Xenogen) and subjected to analysis using Living Image software (Xenogen). Intensities of photon fluxes (photons per s per sr per cm^2^) were then calculated for each mouse with regard to the regions of interest in the thorax, by subtracting the background values of each corresponding area. For histology, lungs were fixed in 10% neutral buffered formalin. Sections were stained with hematoxylin using standard procedures at Morpho Technology.

### Drug resistance *in vitro*

Cells, pretreated with siRNAs or a control oligonucleotide bearing an irrelevant sequence for 24 h, were plated in 96-well culture plates at 3 × 10^3^ cells per well, and drugs were applied on the next day. After incubation for a further 3 days, cell viabilities were measured.

### Drug resistance *in vivo*

All animal experiments were conducted under a protocol approved by the animal care committee of Hokkaido University. 1 × 10^7^ cells, suspended in a mixture of Hank's balanced salt solution (Gibco) and Matrigel (BD BioSciences), were subcutaneously implanted into 4-week-old female nude mice. When tumours grew to ∼50 mm^3^, animals were randomized into two groups (*n*=5 per group). One group was intraperitoneally treated with Temsirolimus (10 mg kg^−1^) and the other with a vehicle control, on a QDx5 schedule for 3 weeks. Tumour volumes were estimated from weekly caliper measurements using the following formula: 0.5 × *L* × *W*^2^. Data were analysed by the Student's *t*-test.

### Patient samples

One hundred and twenty primary ccRCC specimens were obtained from individual patients (93 men and 27 women; mean age: 59 years; range: 25–87 years) who underwent nephrectomy between 1991 and 2003 at Keio University Hospital. None of the patients received chemotherapy or radiation therapy before surgery. The Union for International Cancer Control tumour–node–metastases system was used for tumour staging^37^, and nuclear grading was performed according to the nuclear grading method reported by Fuhrman *et al*^38^. According to the Fuhrman nuclear grade, patients were divided into low grade (grades 1 and 2) or high grade (grades 3 and 4). All patients were followed with clinical and radiological examinations. Clinicopathological parameters of the patients at the time of nephrectomy are summarized in Supplementary Tables 4,5. During the follow-up period, 45 patients developed metastatic disease, and 23 patients died of disease. This study was approved by the Institutional Review Board of Keio University Hospital and informed consent for the experimental use of samples was obtained from the patients according to the hospital's ethical guidelines.

### Immunohistochemistry

Immunohistological analysis was performed as described previously^39^. Briefly, paraffin sections were heated for 15 min in 100 mM Tris-HCl (pH 9.0; for staining of LPAR2, EFA6B and AMAP1) or 10 mM sodium citrate (pH 6.0; for EPB41L5) using a microwave. They were then incubated with a rabbit polyclonal anti-LPAR2 antibody (1:50, HPA019616, Sigma-Aldrich), anti-AMAP1 antibody (1:500, ref. 10), anti-EFA6B antibody (1:50, HPA034722, Sigma-Aldrich), or anti-EPB41L5 antibody (1:1,000). For negative controls, tissues were incubated with non-immune rabbit IgG (Sigma-Aldrich) at the same concentration as that used for each antibody. After washing with PBS, the slides were incubated with anti-rabbit IgG conjugated to peroxidase-labelled dextran polymer (no dilution: EnVision+Rabbit; DAKO Japan) for 15 min, and colour was developed with 3,3'-diaminobenzamine tetrahydrochloride in 50 mM Tris-HCl (pH 7.5) containing 0.005% hydrogen peroxide. The sections were counterstained with hematoxylin. Staining by each antibody often tended to be diffuse. Therefore, the samples showing apparent staining at the cell surface or cytoplasm were classified as those with high staining, and those with negligible or weak staining were classified as low staining. Evaluation of immunostaining was independently carried out by two pathologists (S.M. and Y.O.). Kaplan–Meier analysis was used for the survival data. Overall survival was defined as the interval between surgery and death, or between surgery and the last observation point. For surviving patients, the data were censored at the last follow-up. Progression-free survival was defined as the interval between the date of surgery and the date of diagnosis of any type of relapse. Difference in survival between groups was evaluated by the log-rank test. StatView for Windows version 5.0 (Abacus Concepts) was used to calculate statistical differences between groups.

## Additional information

**How to cite this article:** Hashimoto, S. *et al*. Lysophosphatidic acid activates Arf6 to promote the mesenchymal malignancy of renal cancer. *Nat. Commun.* 7:10656 doi: 10.1038/ncomms10656 (2016).

## Supplementary Material

Supplementary Information Supplementary Figures 1-13 and Supplementary Tables 1-5
