# Supplementary material for: Lysophosphatidic acid activates Arf6 to promote the mesenchymal malignancy of renal cancer
Source: Nat Commun. 2016 Feb 8;7:10656. doi: 10.1038/ncomms10656 (PMC4748122; doi:10.1038/ncomms10656)
Supplement: Supplementary Information — Supplementary Figures 1-13 and Supplementary Tables 1-5 [file ncomms10656-s1.pdf]

Supplementary Information

Supplementary Figure 1

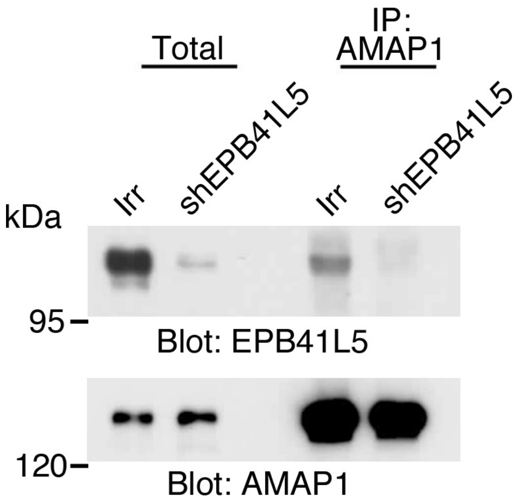

Supplementary Figure 1 | Complex formation of EPB41L5 with AMAP1 in LPA-stimulated 786-O cells Coprecipitation of EPB41L5 with an anti-AMAP1 immunoprecipitants was assessed using 786-O cells, treated with shRNAs for *EPB41L5* or an irrelevant control (Irr), in the presence of LPA. Total: total cell lysates.

Supplementary Figure 2

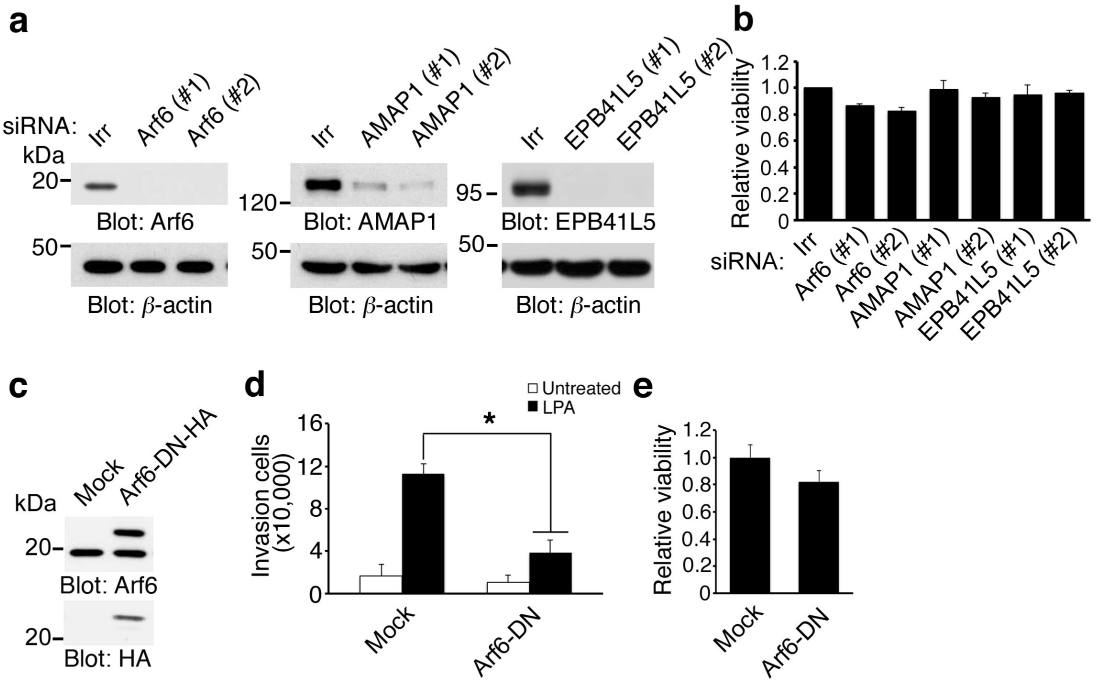

Supplementary Figure 2 | LPA activates Arf6 to promote invasion (**a, b**) 786-O cells were treated with indicated siRNAs; and efficiencies of the silencing (**a**), and viability of the siRNA-treated cells (**b**) are shown. Irr, an oligonucleotide bearing an irrelevant sequence. Protein levels were analyzed by immunoblotting using the indicated antibodies, in which *β*-actin was included as a control (**a**). The results represent the mean ± s.e.m., $n = 3$, in which the viability of cells treated with Irr was normalized to 1.0 (**b**). (**c-e**) Expression of a dominant-negative Arf6 (Arf6-DN) in 786-O cells (**c**) blocks the LPA-induced Matrigel invasion (**d**), without affecting cell viability (**e**). In **d**, error bars show the mean ± s.e.m., $n = 3$. $*P < 0.01$. Statistical analyses were performed using ANOVA.

Supplementary Figure 3

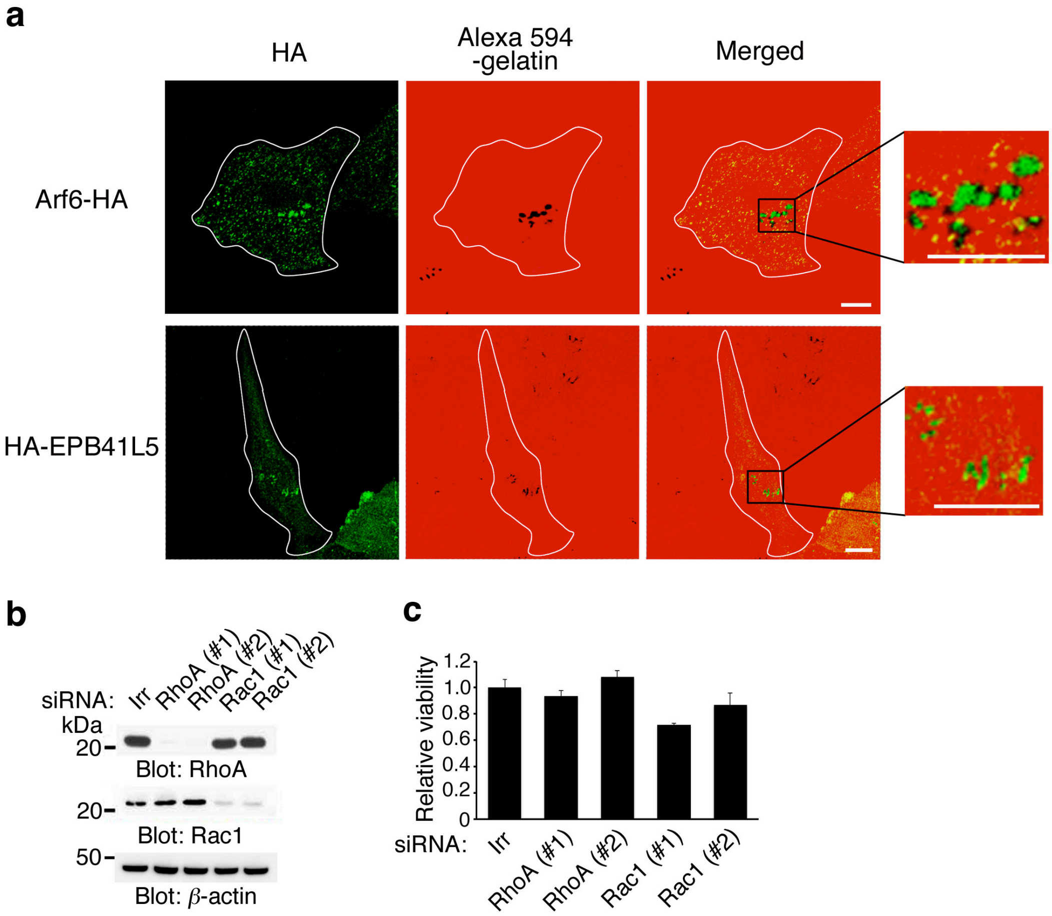

Supplementary Figure 3 | Properties of the LPA-induced invasiveness of 786-O cells (**a**) Accumulation of Arf6 and EPB41L5 at invadopodia. The invadopodia of cells expressing HA-tagged Arf6 or HA-tagged EPB41L5 were formed in the presence of LPA as in Fig. 2a. HA-tagged proteins were labeled using an anti-HA antibody. Bar, 10 µm. (**b**, **c**) siRNA-mediated silencing of *RhoA* and *Rac1*. Efficiencies of the silencing (**b**) and viabilities of the siRNA-treated cells (**c**). siRNA with an irrelevant sequence (Irr) was used as a control. Protein levels were analyzed by immunoblotting using antibodies as indicated, in which *β*-actin was included as a control (**b**). In **c**, error bars represent the mean ± s.e.m., $n = 3$.

Supplementary Figure 4

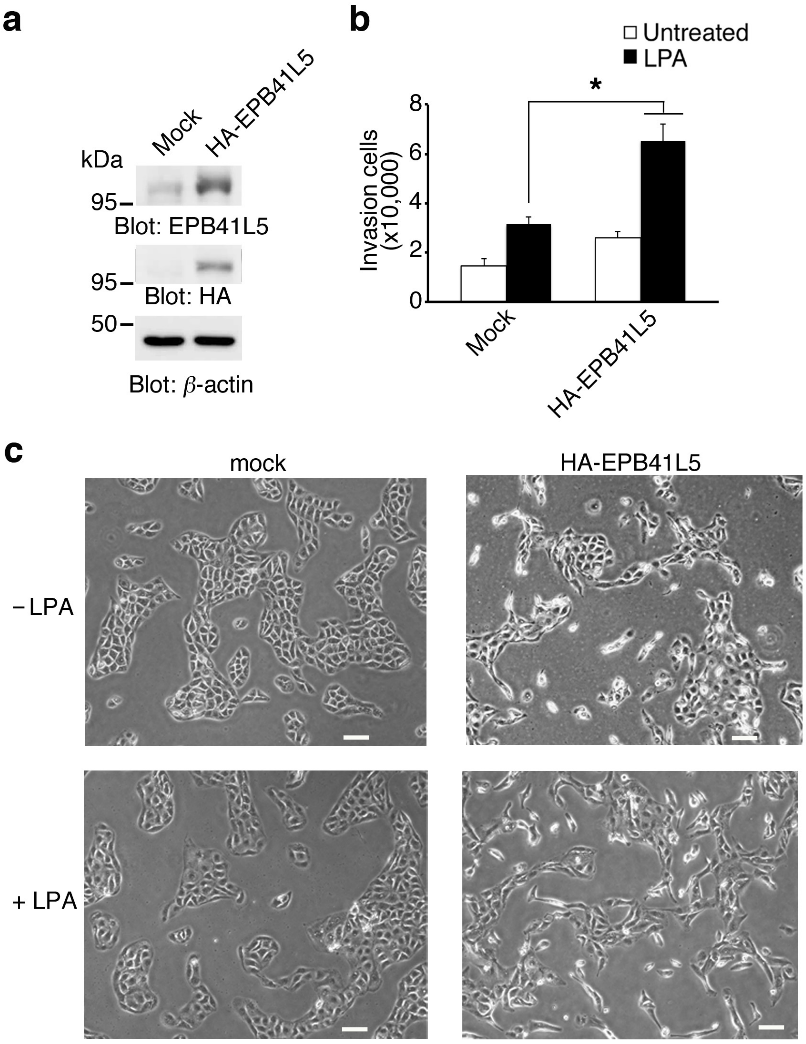

Supplementary Figure 4 | EPB41L5 in invasion of renal cancer cells (**a-c**) Forced expression of EPB41L5 promotes invasiveness of 769-P cells. Expression of HA-EPB41L5 was assessed by immunoblotting using anti-EPB41L5 and anti-HA antibodies (**a**). *β*-actin blot was included as a control. LPA-induced Matrigel invasion activities of 769-P cells, with and without the exogenous EPB41L5 (**b**). Error bars show the mean ± s.e.m., $n = 3$. *$P < 0.05$. Statistical analyses were performed using ANOVA. Morphology of 769-P cells, with or without the exogenous EPB41L5, after cultured with or without LPA for 20 h (**c**). Scale bar, 100 μm.

Supplementary Figure 5

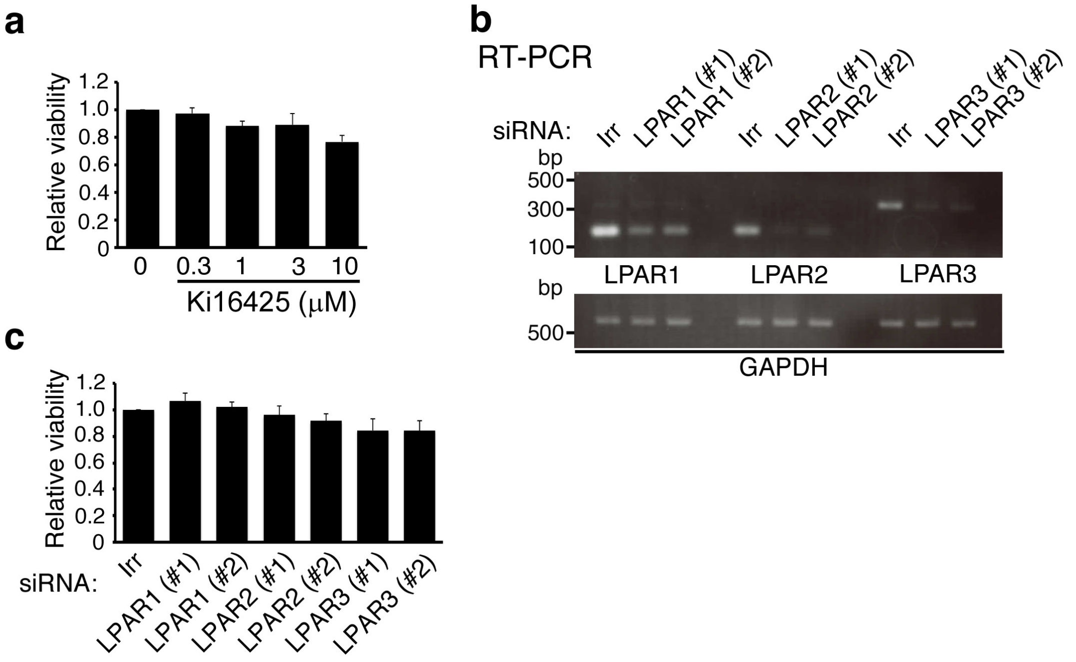

Supplementary Figure 5 | Involvement of LPARs in invasion (**a**) Viability of 786-O cells treated with Ki16425 at concentrations as indicated, or with DMSO (0) for 20 h. (**b**, **c**) Efficiency of the siRNA-mediated silencing of genes encoding LPARs, as assessed by RT-PCR (**b**), and viability of the siRNA-treated cells (**c**). *GAPDH* was included as a control (**b**). In **a** and **c**, error bars show the mean ± s.e.m., $n = 3$.

Supplementary Figure 6

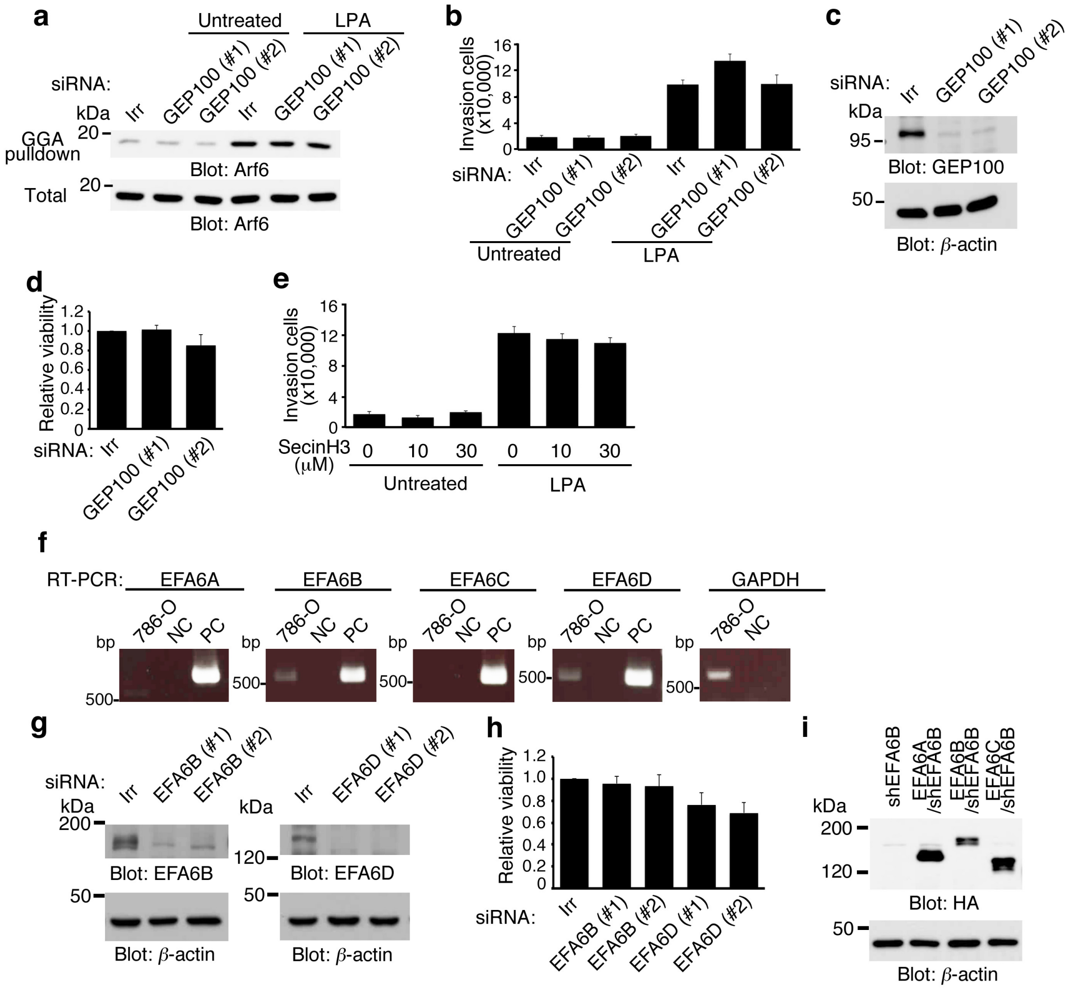

Supplementary Figure 6 | Requirement for EFA6 but not GEP100 in the LPA-induced invasion (**a-d**) *GEP100* silencing did not affect LPA-induced Arf6 activation (**a**) and Matrigel invasion (**b**) of 786-O cells. Efficiency of the siRNA treatment on *GEP100* protein levels (**c**) and cell viability (**d**) are also shown. (**e**) SecinH3 does not affect Matigel invasion of 786-O cells. SecinH3 was applied to cells 3 h prior to the experiments and was present during the experiments at concentrations as indicated. Controls included cells treated with DMSO (0). (**f**) 786-O cells express *EFA6B* and *EFA6D* mRNAs endogenously. mRNA expression of the EFA6 isoforms in

786-O cells was analyzed by RT-PCR. Two nanograms of cDNAs for each of the indicated genes was used as a positive control (PC). NC, without template cDNAs. *GAPDH* was included as a control. (**g**, **h**) Efficiency of siRNA-mediated silencing of *EFA6B* and *EFA6D* in 786-O cells (**g**), and the viability of the siRNA-treated cells (**h**). (**i**) Exogenous expression of EFA6A-C in 786-O cells, in which endogenous EFA6B was silenced. shEFA6B, endogenous EFA6B-silenced cells transfected with an empty vector. A control included a β-actin immunoblot. In **a**-**d**, **g**, and **h**, Irr, an oligonucleotide bearing an irrelevant sequence. In **b, d, e,** and **h**, error bars show the mean ± s.e.m., $n = 3$.

Supplementary Figure 7

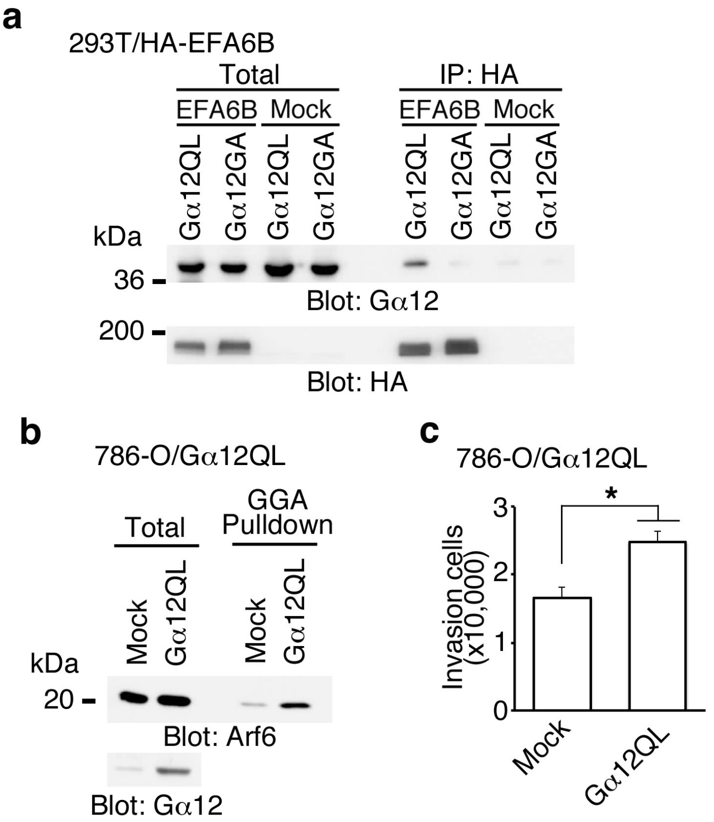

Supplementary Figure 7 | The dominant-active form of Gα12 binds to EFA6 and activates Arf6 (**a**) EFA6B binds to the GTP-hydrolysis deficient dominant-active form of Gα12, namely Gα12QL, but not its GTP-binding defective dominant-negative mutant, Gα12GA. Gα12QL and Gα12GA were expressed in 293T cells together with HA-EFA6B, by cDNA transfection, and their co-precipitation was examined by immunoprecipitation using anti-HA antibody, coupled with immunoblotting as indicated. Mock, an empty vector was transfected instead of the HA-EFA6B cDNA. (**b**, **c**) Gα12QL activates Arf6 and promotes cell invasion without LPA stimulation. Arf6 activities (**b**) and the Matrigel invasion activities (**c**) were measured with unstimulated 786-O cells, transfected with the Gα12QL cDNA or an empty vector (Mock). In **c**, error

bars show the mean ± s.e.m., $n = 3$. *$P < 0.05$. Statistical analyses were performed using ANOVA.

Supplementary Figure 8

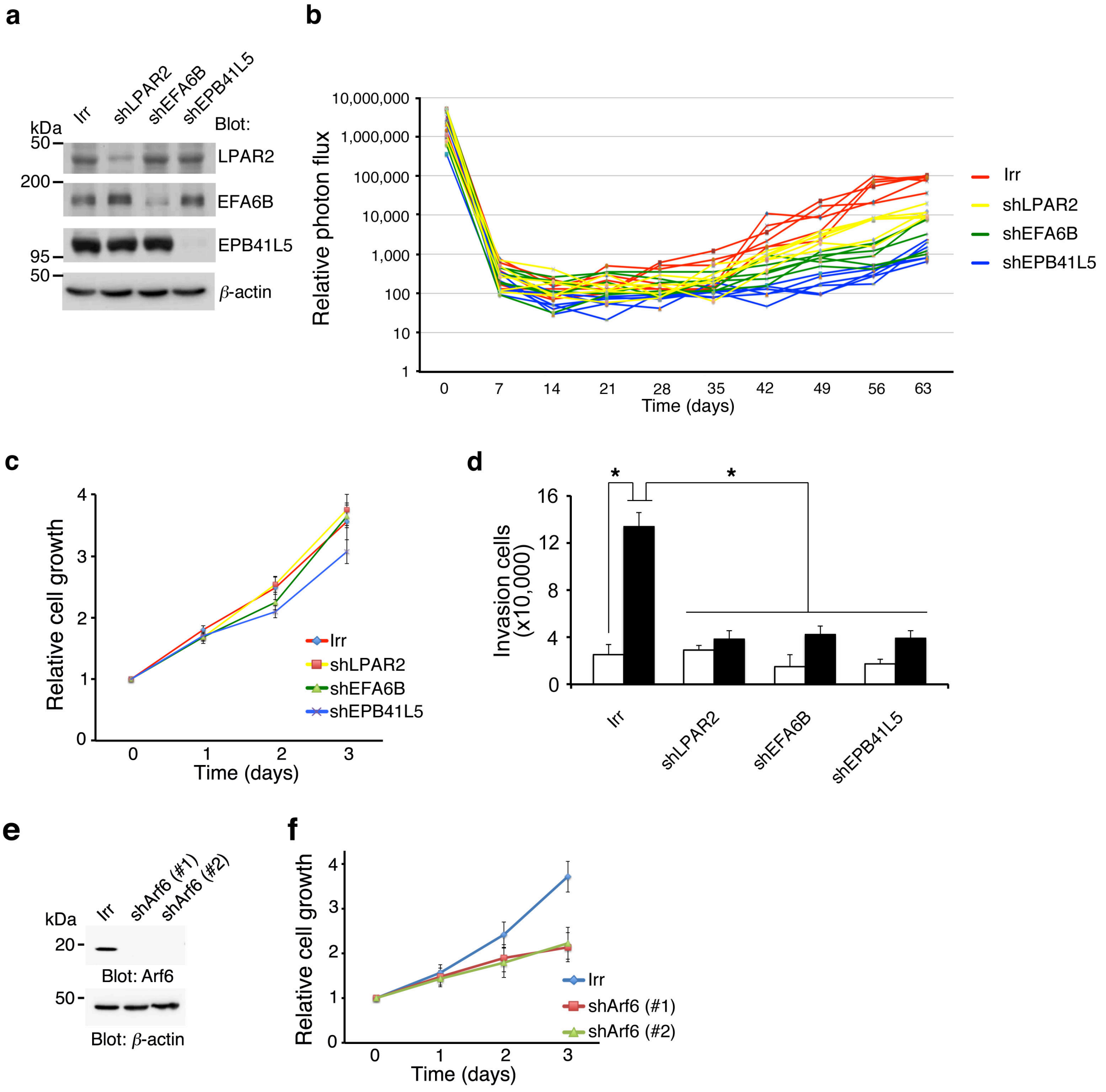

Supplementary Figure 8 | Critical roles of LPAR2 and the Arf6 mesechymal pathway in the lung metastasis (**a**) Efficiency of shRNA-mediated silencing of the indicated genes in 786-O cells expressing a luciferase reporter, as assessed by the immunoblotting of cell lysates using the indicated antibodies. Cells treated with shRNA plasmids bearing an irrelevant sequence (Irr) were included as a control. *β*-actin immunoblot was included as a control. (**b**) Time-course records of the growth of shRNA-treated 786-O cells in individual mice. Bioluminescence intensities of the

chests of individual mice, injected with shRNA-treated cells or the control cells (Irr), were measured on the indicated days post injection. (**c, d**) *In vitro* proliferation (**c**) and Matrigel invasion (**d**) of 786-O cells transfected by indicated shRNAs. (**e, f**) Efficiency of shRNA-mediated silencing of *Arf6* in 786-O cells (**e**) and the *in vitro* proliferation of these cells (**f**). In **c, d,** and **f**, error bars show the mean ± s.e.m., $n = 3$. $*P < 0.01$. Statistical analyses were performed using ANOVA.

Supplementary Figure 9

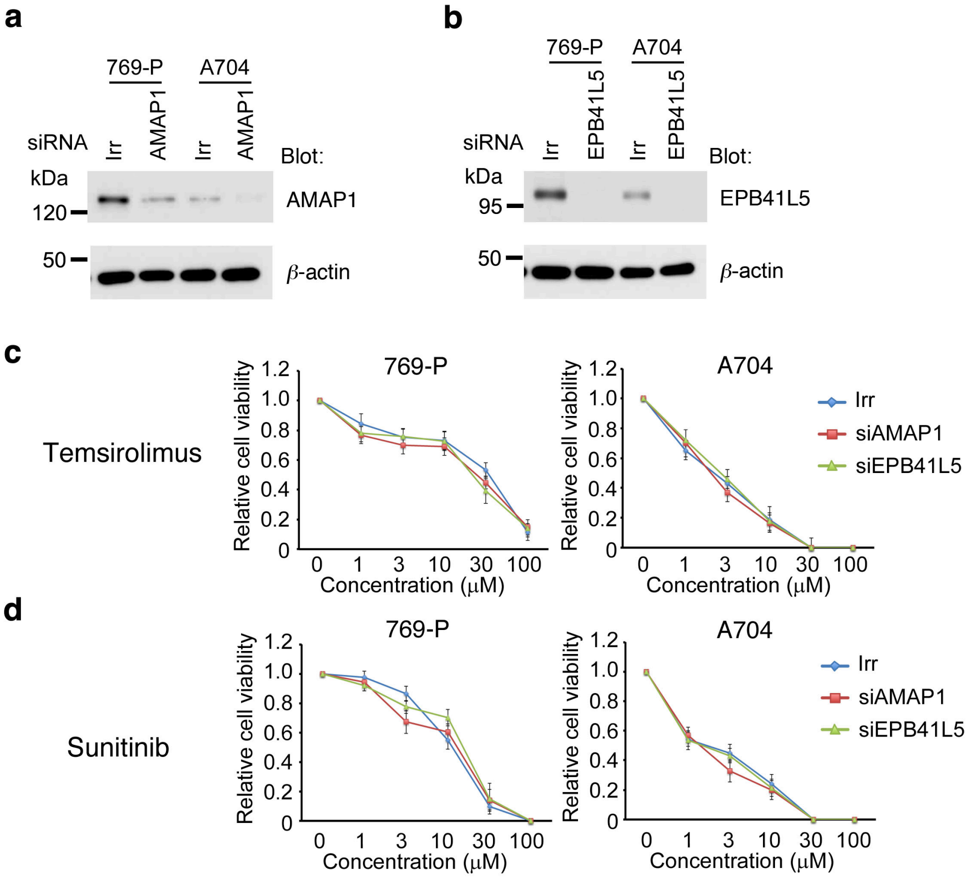

Supplementary Figure 9 | Effects of the silencing of *AMAP1* and *EPB41L5* on drug resistance of different renal cancer cell lines (**a**, **b**) Efficiency of siRNA-mediated silencing of AMAP1 (**a**) and EPB41L5 (**b**) expression, as assessed by immunolotting by their antibodies, in 769-P cells and A704 cells. *β*-actin immunoblots were included as controls. Irr, an oligonucleotide bearing an irrelevant sequence. (**c**, **d**) No significant effects of siRNA-mediated silencing of *AMAP1* and *EPB41L5* on the resistance of 769-P cells and A704 cells to Temsirolimus (**c**) and Sunitinib (**d**). In **c** and **d**, drug resistance was assessed as in Fig. 7. Data are presented as ratios calculated by normalizing the values obtained from the untreated cells as 1.0. Error bars show the mean ± s.e.m., $n = 3$.

Supplementary Figure 10

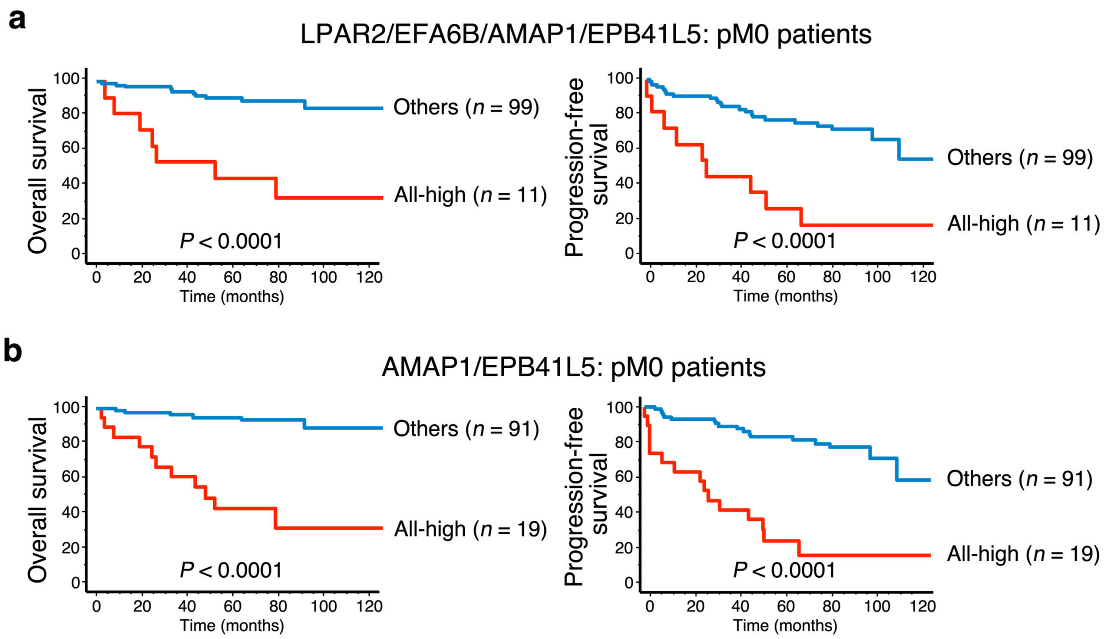

Supplementary Figure 10 | The Arf6-based mesenchymal pathway in poor clinical outcome of the pM0 patients. (**a, b**) Kaplan–Meier curves of the overall survival and progression-free survival of ccRCC patients, who did not have metastasis at time of the diagnosis ($n = 110$), with regard to the high expression of all components (**a**), and with regard to the simultaneous high expression of AMAP1 and EPB41L5 (**b**), as indicated. *P*-values represent the results of the log-rank test.

Supplementary Figure 11

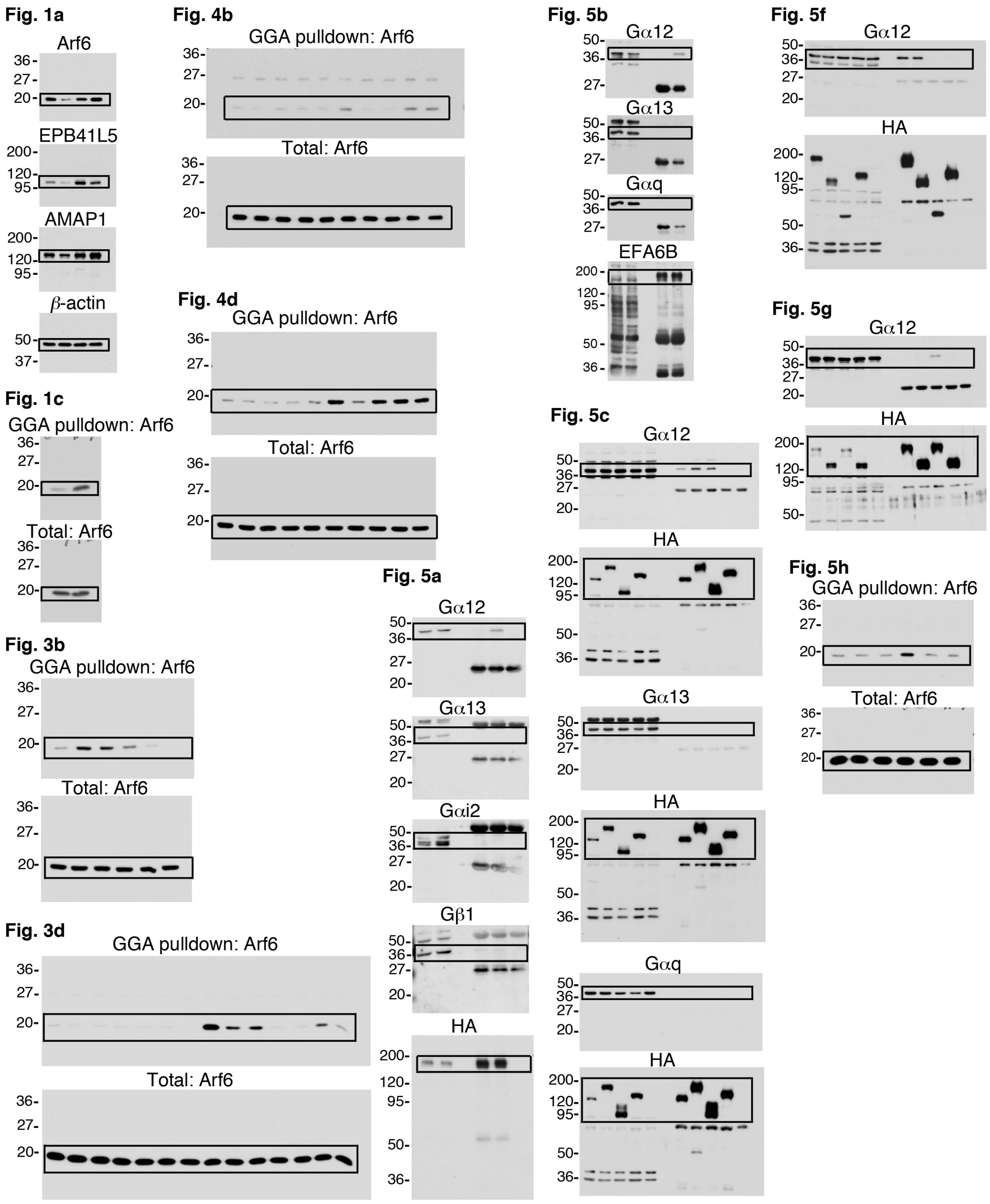

Supplementary Figure 11 | Uncropped images of blots shown in Figures 1, 3, 4, and 5

Supplementary Figure 12

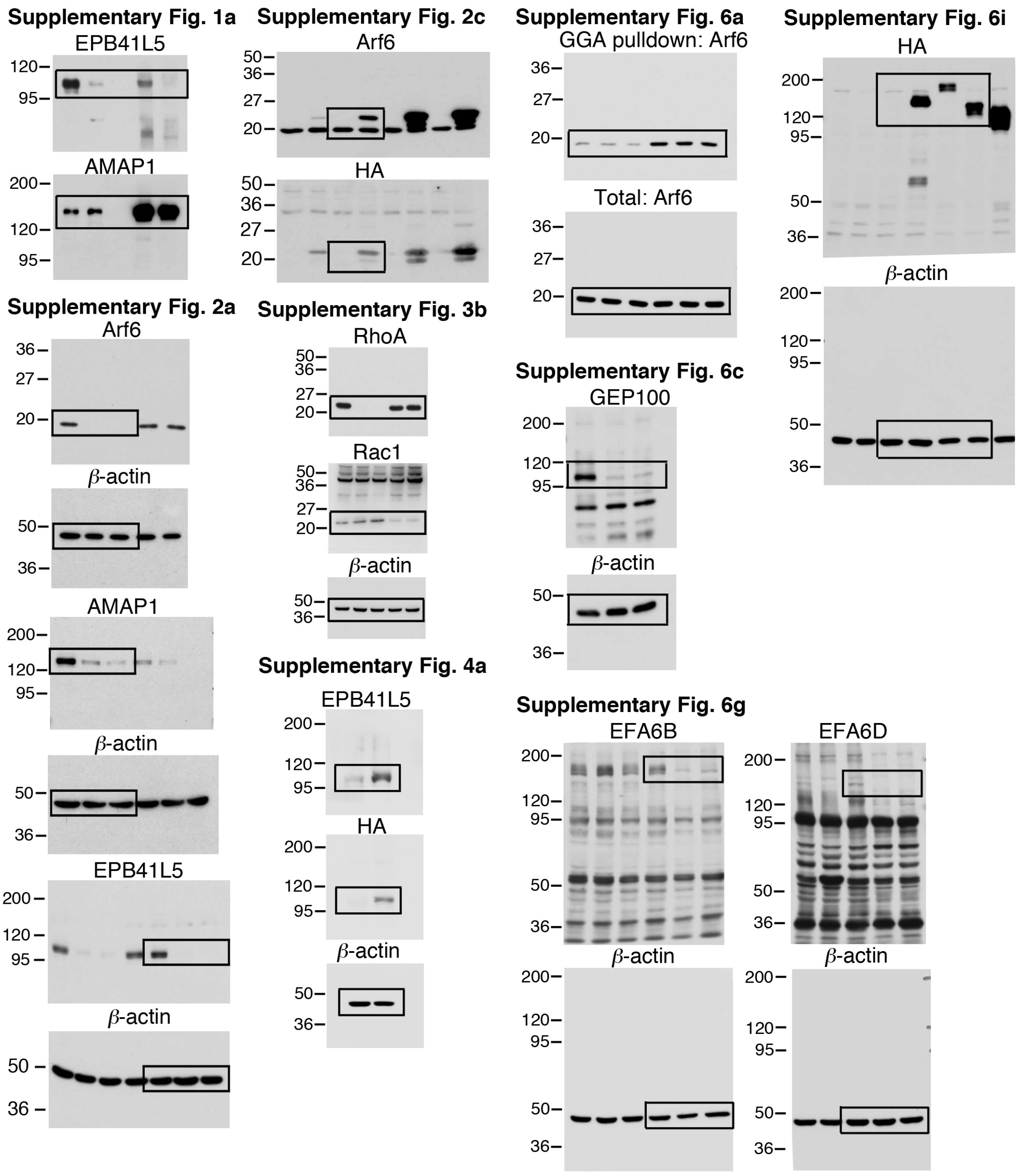

Supplementary Figure 12 | Uncropped images of blots shown in Supplementary Figures 1, 2, 3, 4, and 6

Supplementary Figure 13

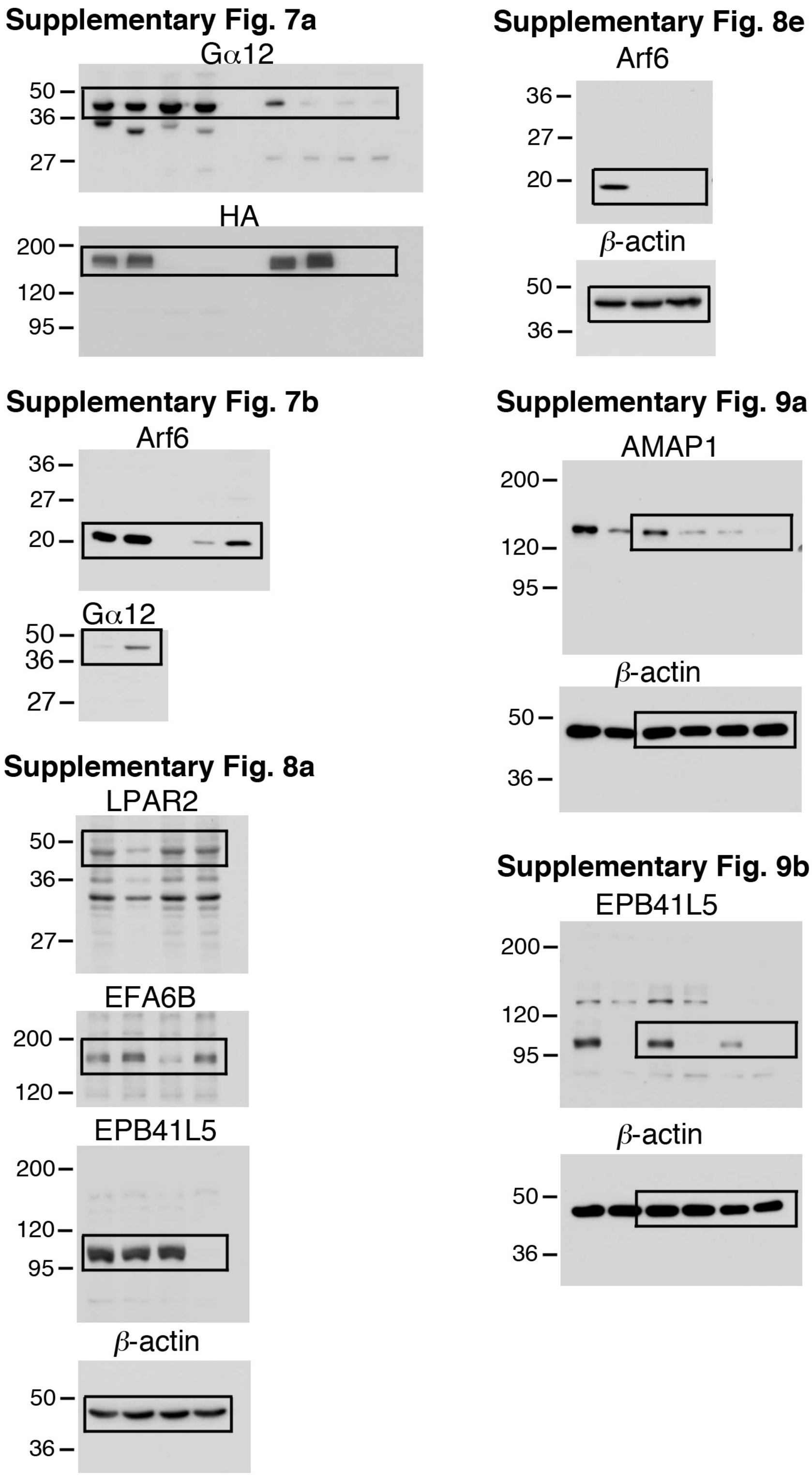

Supplementary Figure 13 | Uncropped images of blots shown in Supplementary Figures 7, 8, and 9

Supplementary Table 1 | Oligonucleotides for cDNA cloning

	Sequence (5'-3')
EFA6A FL Fw	GTCGCGGCCGCATGGCCCAGGGTGCCATGC
EFA6A FL Rv	GTCGCGGCCGCTCAGGGCTTCCGCCGCCCA
EFA6B FL Fw	GTCGCGGCCGCATGATGGGTGACTACAGA
EFA6B FL Rv	GTCGCGGCCGCTCACAGCTGATTGCGGTTC
EFA6B N-terminal Fw	GTCGCGGCCGCATGATGGGTGACTACAGA
EFA6B N-terminal Rv	GTCGCGGCCGCTCAGCCTTCCTCTGGGGAC
EFA6B C-Terminal Fw	GTCGCGGCCGCCAGAGACCACCAGCTGGAG
EFA6B C-Terminal Rv	GTCGCGGCCGCTCACAGCTGATTGCGGTTC
EFA6B ΔRGS Fw	AAACCACACTCCATCTGCTGGGCCTCAGTG
EFA6B ΔRGS Rv	TGCTGTGTTCTGCCTGTGCTCCCTCTGGGCAG
EFA6C FL Fw	GTCGCGGCCGCATGGAGGAGGACAAGCTCT
EFA6C FL Rv	GTCGCGGCCGCCTAAGTATCAGGCCCAGTG
EFA6D FL Fw	GTCGCGGCCGCATGGAAGGAAGGAGCGCAGCGGCAGAG
EFA6D FL Rv	TAGCGGCCGCCTAAGTAACTTTTTGCTTAAT
EPB41L5 FL Fw	ATAGCGGCCGCATGCTGAGTTTCTTCCGTAG
EPB41L5 FL Rv	ATGCGGCCGCTCAGAGCTCAGTGGTCAGTAAACA
Gα12 FL Fw	ATGGATCCGCCACCATGTCCGGGGTGGTGCGGACCCTC
Gα12 FL Rv	ATGCTAGCTCACTGCAGCATGATGTCCTTCAG
Gα12 G230A Fw	GTGGATGTGGGCGCCCAGCGGTCCCAGCGC
Gα12 G230A Rv	CATCTTAAAGGGGATCTTCTTAATAACGAAGTC

Supplementary Table 2 | Target sequences for RNA interference

siRNA

	target sequence (5'-3')
Arf6 #1	AAGCACCGCAUUAUCAAUGACCG
Arf6 #2	CAACGUGGAGACGGUGACUU
AMAP1 #1	AAGACCUGACAAAAGCCAUUA
AMAP1 #2	CCAGGGAUUUACUUGCACUAA
EPB41L5 #1	GAGAUGGAACUGGCUAUUUUU
EPB41L5 #2	UUCAGAUUCGUGCCUAUUCAG
GEP100 #1	AAGUGAAAUCACUGGCCGAG
GEP100 #2	CCAGUACCAGAUGAACAAGAA
LPAR1 #1	UUGCAAUCGAGAGGCACAUUA
LPAR1 #2	GUUCAACACAGGACCCAAUAC
LPAR2 #1	CCUGGUCAAGACUGUUGUCAU
LPAR2 #2	GCGAGUCUGUCCACUAUACAU
LPAR3 #1	GCCGAUUCUUCGCUGGAAUU
LPAR3 #2	GCCUAUGUAUUCCUGAUGUUU
EFA6B #1	CAGCCAAGUUCCUUGAAGAAA
EFA6B #2	CACCUUGACAUGUGCAAUCAU
EFA6D #1	AACCGGAUUCUGGUACCAGCU
EFA6D #2	AACCUGGAGGGCCUCAAUGAU

shRNA

	target sequence (5'-3')
LPAR2	TRCN0000011375: CCTGGTCAAGACTGTTGTCAT
EFA6B	TRCN0000427083: TCCCTGGAGGAGACTTATTTC
EPB41L5	TRCN0000130203: CCTACCATGTATGAAGCTATA
Arf6 (#1)	TRCN0000294069: CTTGCTGTAGATGGCTTATTT
Arf6 (#2)	TRCN0000048003: GTCAAGTTCAACGTATGGGAT

Supplementary Table 3 | Primers for RT-PCR

	Sequence (5'-3')
LPAR1 Fw	GTGTGGGCTGGAACTGTATCTG
LPAR1 Rv	TAGTCCTCTGGCGAACATAG
LPAR2 Fw	GGCCAGTGCTACTACAACGAGACC
LPAR2 Rv	TGGAGGCGATGGCTGCTATGAC
LPAR3 Fw	CCTGGTGGTTCTGCTCCTCGAC
LPAR3 Rv	GTGCCATACATGTCCTCGTCCTTG
EFA6A Fw	AGCCAGCTGGTGTCCGACTCA
EFA6A Rv	GGAGCTAACAGCAGCTGGGAA
EFA6B Fw	GAGGACACCGATGAACTCTTC
EFA6B Rv	GTGTCTTCTTCATCCACGG
EFA6C Fw	GGATGGCCTGTCAGACTCAGA
EFA6C Rv	CCTCAGCTCATCCTCATCAATGG
EFA6D Fw	CAACACGGCTAGAAGCTCATT
EFA6D Rv	TTCATCATCTACTGCCCATTC
GAPDH Fw	ACCCAGAAGACTGTGGATGG
GAPDH Rv	TCTAGACGGCAGGTCAGGTC

Supplementary Table 4 | Summary of clinicopathological features in 120 clear cell renal cell carcinomas and their association with the expression of LPAR2, EFA6B, AMAP1, and EPB41L5

Feature	LPAR2-high % (*n*)	EFA6B-high % (*n*)	AMAP1-high % (*n*)	EPB41L5-high % (*n*)
All patients (n = 120)	56 (67)	42 (50)	35 (42)	33 (40)
Sex				
Male (*n* = 93)	57 (53)	41 (38)	34 (32)	35 (33)
Female (*n* = 27)	52 (14)	44 (12)	37 (10)	26 (7)
P	0.665	0.826	0.822	0.487
Age at surgery				
< 59 years (*n* = 59)	47 (28)	37 (22)	29 (17)	36 (21)
≥ 59 years (*n* = 61)	15 (9)	46 (28)	41 (25)	31 (19)
P	0.098	0.360	0.184	0.699
Pathological tumor stage				
pT1, 2 (*n* = 86)	43 (37)	31 (27)	21 (18)	23 (20)
pT3, 4 (*n* = 34)	88 (30)	68 (23)	71 (24)	59 (20)
P	< 0.001	< 0.001	< 0.001	< 0.001
Lymph node metastasis				
pN0 (*n* = 115)	54 (62)	39 (45)	33 (38)	32 (37)
pN1,2 (*n* = 5)	100 (5)	100 (5)	80 (4)	60 (3)
P	0.066	0.011	0.050	0.332
Distant metastasis				
pM0 (*n* = 110)	53 (58)	38 (42)	32 (35)	31 (34)
pM1 (*n* = 10)	90 (9)	80 (8)	70 (7)	60 (6)
P	0.041	0.016	0.032	0.082
Histological grade				
G1, 2 (*n* = 81)	43 (35)	27 (22)	19 (15)	19 (15)
G3, 4 (*n* = 39)	82 (32)	72 (28)	69 (27)	64 (25)
P	< 0.001	< 0.001	< 0.001	< 0.001

Supplementary Table 5 | Summary of clinicopathological features in 120 clear cell renal cell carcinomas and their association with the co-expression of LPAR2, EFA6B, AMAP1 and EPB41L5

Feature	LPAR2/EFA6B/AMAP1/EPB41L5-high % (*n*)	AMAP1/EPB41L5-high % (*n*)
All patients (*n* = 120)	13 (15)	20 (24)
Sex		
Male (*n* = 93)	14 (13)	20 (19)
Female (*n* = 27)	7 (2)	19 (5)
P	0.517	> 0.999
Age at surgery		
< 59 years (*n* = 59)	15 (9)	22 (13)
≥ 59 years (*n* = 61)	10 (6)	18 (11)
P	0.418	0.652
Pathological tumor stage		
pT1, 2 (*n* = 86)	5 (4)	9 (8)
pT3, 4 (*n* = 34)	32 (11)	47 (16)
P	< 0.001	< 0.001
Lymph node metastasis		
pN0 (*n* = 115)	10 (12)	18 (21)
pN1,2 (*n* = 5)	60 (3)	60 (3)
P	0.014	0.054
Distant metastasis		
pM0 (*n* = 110)	10 (11)	17 (19)
pM1 (*n* = 10)	80 (4)	50 (5)
P	0.022	0.027
Histological grade		
G1, 2 (*n* = 81)	4 (3)	5 (4)
G3, 4 (*n* = 39)	31 (12)	51 (20)
P	< 0.001	< 0.001
